# The Evidence-Base for Psychodynamic Psychotherapy With Children and Adolescents: A Narrative Synthesis

**DOI:** 10.3389/fpsyg.2021.662671

**Published:** 2021-04-27

**Authors:** Nick Midgley, Rose Mortimer, Antonella Cirasola, Prisha Batra, Eilis Kennedy

**Affiliations:** ^1^Research Department of Clinical, Educational and Health Psychology, University College London, London, United Kingdom; ^2^Child Attachment and Psychological Therapies Research Unit (ChAPTRe), Anna Freud National Centre for Children and Families, London, United Kingdom; ^3^Research and Development Unit, Tavistock and Portman NHS Trust, London, United Kingdom

**Keywords:** child and adolescent psychotherapy, evidence based practice, psychodynamic psychotherapy, systematic review, effectiveness and efficacy

## Abstract

Despite a rich theoretical and clinical history, psychodynamic child and adolescent psychotherapy has been slow to engage in the empirical assessment of its effectiveness. This systematic review aims to provide a narrative synthesis of the evidence base for psychodynamic therapy with children and adolescents. Building on two earlier systematic reviews, which covered the period up to 2017, the current study involved two stages: an updated literature search, covering the period between January 2017 and May 2020, and a narrative synthesis of these new studies with those identified in the earlier reviews. The updated search identified 37 papers (28 distinct studies). When combined with papers identified in the earlier systematic reviews, this resulted in a combined total of 123 papers (82 distinct studies). The narrative synthesis of findings indicates that there is evidence of effectiveness for psychodynamic therapy in treating a wide range of mental health difficulties in children and adolescents. The evidence suggests this approach may be especially effective for internalizing disorders such as depression and anxiety, as well as in the treatment of emerging personality disorders and in the treatment of children who have experience of adversity. Both the quality and quantity of empirical papers in this field has increased over time. However, much of the research demonstrates a range of methodological limitations (small sample sizes, lack of control groups etc.), and only 22 studies were Randomized Controlled Trials. Further high-quality research is needed in order to better understand the effectiveness of psychodynamic psychotherapy for children and young people.

## Introduction

Despite the rich theoretical and clinical history, psychodynamic child and adolescent psychotherapy has been slow to engage with issues regarding the evaluation of treatment outcomes (Midgley, 2009)^1^. As the philosophy of evidence-based practice has evolved, child psychotherapists have increasingly accepted the importance of evaluating the effectiveness of their work, but often lack the skills and competencies—or the funding (MQ, 2017)—to carry out the necessary research. It is within this context that a first review of the evidence for psychodynamic child psychotherapy was commissioned in the UK (Kennedy, 2004). This ground-breaking review identified 32 papers, reporting on 32 distinct research studies, that set out to evaluate the effectiveness of different types of psychodynamic child therapy for different populations. Although the findings of this review were promising, only five of the studies were randomized controlled trials (RCTs).

Building on the findings of this first systematic review, an update which incorporated the earlier findings was published in 2011 (Midgley and Kennedy, 2011) and a further update was published in 2017 (Midgley et al., 2017). Other reviews of the evidence-base, using slightly different inclusion criteria and search strategies, have also been carried out (e.g., Abbass et al., 2013; Palmer et al., 2013). The Abbass et al. (2013) review was especially important because, for the first time, it took a meta-analytic approach, which goes some way toward addressing the problem of low statistical power that has been a problem for child psychotherapy research to date. Although including a smaller number of studies (11) and focusing only on short-term psychodynamic psychotherapy (STPP) for adolescents, all studies included were clinical trials. The meta-analysis demonstrated robust (g = 1.07, 95% CI: 0.80–1.34) within group effect sizes, suggesting the treatment may be effective. These effects further increased in follow up compared to post treatment (overall, g = 0.24, 95% CI: 0.00–0.48). When compared to a range of other treatments, such as CBT or systemic family therapy, child psychotherapy showed comparable effectiveness.

Although this series of systematic reviews has played an important role in bringing together the evidence-base for psychodynamic child and adolescent psychotherapy, these earlier reviews each covered only a set period (pre-2011, or 2011–2017), or a certain sub-set of studies (such as clinical trials of short-term therapy for adolescents) and did not provide a synthesis of all of outcome research related to psychodynamic child and adolescent psychotherapy to date. Given the rapid developments in this field, the aim of this review was to provide an update on the evidence base for psychodynamic therapy with children and adolescents published between January 2017 and May 2020, including an assessment of the quality of research done in this area. In addition, this paper provides, for the first time, a narrative synthesis of all the published research to date, synthesizing the findings of this new update (2017–2020) with those reported in the 2011 and 2017 reviews.

The findings of this narrative synthesis will be presented in relation to children and adolescents with different clinical presentations, as well as reviewing the evidence for psychodynamic therapy in “real world” settings, when offered to children with a mix of presenting problems.

## Materials and Methods

The search strategy and methods used in this review mostly follow those of the previous reviews (see Midgley and Kennedy, 2011), with some small changes. Key psychology and psychiatry databases were searched for publications between January 2017 and May 2020. Search terms (see Supplementary Table 1) were derived using the method outlined by Schardt et al. (2007). Inclusion and Exclusion criteria are displayed in Table 1. Additional searching was also undertaken, including contacting key researchers in the field, and hand searching the reference list of relevant papers and reviews.

**Table 1:** Inclusion and Exclusion Criteria.

**Inclusion criteria:**
*Language:* English
*Intervention:* Individual or dyadic (parent-child) psychodynamic and/or psychoanalytic therapy, including family or group therapy where the therapeutic intervention is described as psychodynamic or psychoanalytic. As psychodynamic treatments are based on a range of theories, this review included all studies where the researchers defined the treatment model under investigation as primarily psychodynamic or psychoanalytic
*Participant age:* Studies where a majority of participants were aged between 3 and 18 years old but none of the child/adolescent participants were over 25
*Study focus:* Studies primarily concerned with evaluating treatment outcomes, using any design involving quantitative measurement of outcomes (e.g., randomized control trials, quasi-experimental studies, and naturalistic evaluation)
*Study outcomes:* Outcomes related to any mental health condition or problem, including sub-threshold mental health conditions and prevention of mental health difficulties
**Exclusion criteria:**
*Method:* Studies that report only on qualitative findings; single case studies; review papers; and meta-analyses
*Outcomes:* Studies where child outcomes are not reported (e.g., only parent outcomes reported) and studies focusing only on the process rather than outcome of therapy
*Interventions:* Parent-infant psychotherapy (where the intervention is primarily focused on therapeutic work with children under 3 years of age)

### Data Extraction and Quality Assessment

Studies that met inclusion criteria for the update of this review were summarized and are presented in a data extraction table (see Supplementary Table 2). Where multiple papers described secondary analysis from the same study, papers were grouped together. Studies were sorted by methodology into four groups: randomized controlled trials (RCTs), quasi-experimental studies, observational studies with a comparison control, and observational studies without a control group. Studies were also grouped by presenting problem, such as “depression,” “emerging personality disorders” or “mixed.”

A critical appraisal of each study was then undertaken (see Table 2). Two separate quality assessment tools, designed by the National Institute for Health Research, were used: one for controlled intervention studies, and one for naturalistic pre-post studies without a control group (National Institutes of Health, 2014). The two tools assess the “internal validity” of the study (i.e., to what extent the study contain a risk of bias). To ensure a consistent approach to the risk of bias assessment, one controlled and one non-controlled study were selected, and three authors separately rated these studies using the relevant quality assessment tools. These three authors then met together to discuss any disagreement and reach consensus on how to apply the criteria, before separately rating the remaining papers. Any uncertainties regarding rating of the remaining papers was brought back to the group, and a consensus was reached on the appropriate rating.

**Table 2:** Studies 2017–2020 grouped by Internal Validity (Risk of Bias) Rating.

**Studies rated using the NIHR tool for Controlled Intervention Studies**	**Internal Validity Rating**	**Studies rated using the NIHR tool for Pre-Post Studies with no Control Group**	**Internal Validity Rating**
Cropp et al. (2019)	High	Gatta et al. (2019)	High
Beck et al. (2020); Jørgensen et al. (2020)	High	Pernebo et al. (2018)	High
Lindqvist et al. (2020)	High	Hauber et al. (2017)	High
Goodyer et al. (2017); Davies et al. (2020); O'Keeffe et al. (2020); Reynolds et al. (2020); O'Keeffe et al. (2019); Aitken et al. (2020)	High	Halfon and Bulut (2019); Halfon et al. (2019a,b)	High
Midgley et al. (2019)	High/Medium	Strangio et al. (2017)	High/Medium
Salzer et al. (2018)	High/Medium	Levy (2017)	Medium
Stefini et al. (2017)	High/Medium	Polek and McCann (2020)	Medium
Griffiths et al. (2019)	High/Medium	Chirico et al. (2019)	Medium
Hertzmann et al. (2017)	Medium	Midgley et al. (2018)	Medium/Low
Edginton et al. (2018)	Medium	Bo et al. (2017)	Medium/Low
Krischer et al. (2020)	Medium/Low	Bo et al. (2019)	Medium/Low
Weitkamp et al. (2017)	Medium/Low	Schenk et al. (2019)	Medium/Low
Weitkamp et al. (2018)	Medium/Low	Prout et al. (2019)	Medium/Low
Enav et al. (2019)	Medium/Low	Ryan and Jenkins (2020)	Low
Bernstein et al. (2019)	Low		

*Where a study is rated as having “high internal validity” this means that the outcome results reported in the study have a greater probability of being truly attributed to the intervention or exposure being evaluated, and not to biases, measurement errors, or other confounding factors that may result from flaws in the design or conduct of the study*.

### Data Synthesis

The data extraction table for the studies published since January 2017 was merged with data extraction for the previous two reviews, and the full set of papers was grouped by presenting problem. Given the heterogeneity of study designs, populations and measures, a meta-analytic approach was not appropriate, so findings were synthesized thematically in relation to the primary presenting problems of the children and adolescents in each study. Findings are presented in a narrative form, with only the most significant and/or more recent studies in relation to each clinical group described in more detail; additional information about other studies, grouped by presenting problem, can be found in Supplementary Table 3.

## Results

**Figure 1:**
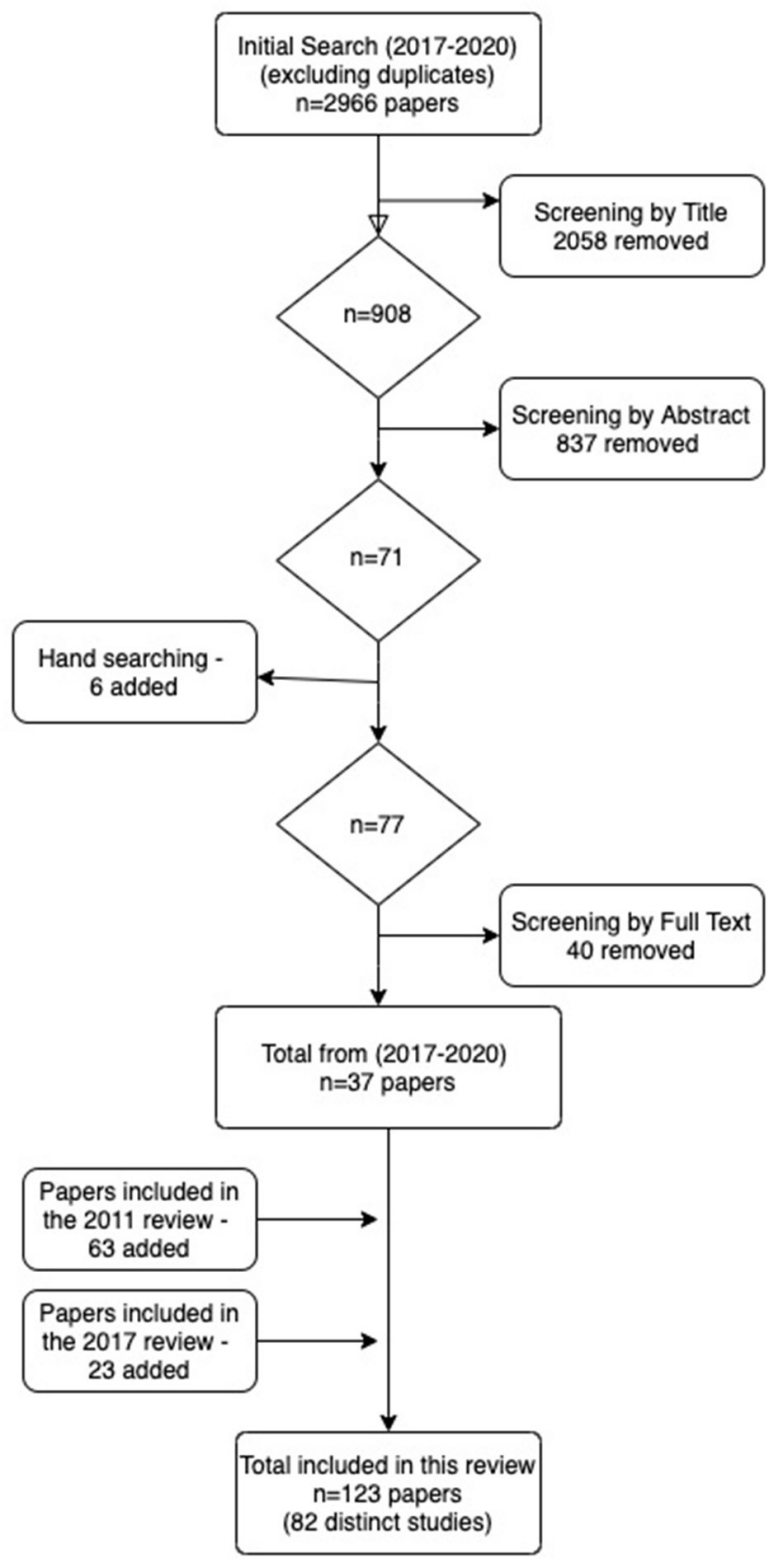
PRISMA flow diagram of the study selection process.

As displayed in Figure 1, in total, 37 papers, were identified in this updated review for the period from January 2017 to May 2020, comprising 28 distinct studies.

Having completed the data extraction and quality assessment of these new studies, the papers were then combined with the papers identified in the previous reviews published in 2011 and 2017 (see Figure 1). This led to a total of 123 papers, comprising 82 distinct studies. Although each study included slightly different age groups, we have used the term “children” to refer primarily to those aged 3–11, and “adolescents” to refer to those aged 12–25 (although in nearly all cases the maximum age was 18).

### Emotional Disorders

Emotional disorders are the most common reason for children and young people to access mental health services. Emotion disorders are relatively common in children; in the UK one in 12 (8.1%) children aged between 5 and 19 have an emotional disorder, and rates are higher for girls (10.0%) than boys (6.2%) (Sadler et al., 2018).

This review identified 24 studies evaluating the psychodynamic treatment of children with a range of emotional disorders: 5 studies focused on mixed emotional disorders, 4 on depression, 2 on self-harm, 6 on anxiety disorders, and 5 on feeding and eating disorders. Additionally, one paper reports secondary analyses from the Anna Freud Retrospective study of a mixed population, focusing on those children diagnosed with emotional disorders.

A number of the earliest evaluations of psychodynamic therapy for children focused on the treatment of emotional disorders (e.g., Smyrnios and Kirby, 1993; Sinha and Kapur, 1999). For example, an Italian quasi-randomized trial (Muratori et al., 2002, 2003, 2005) of time-limited psychodynamic psychotherapy for children aged 6–11 years with emotional disorders demonstrated the potential effectiveness of this treatment for internalizing problems, although outcomes were better when those children had what were considered “pure” rather than “mixed” emotional disorders. Interestingly, children who were offered psychodynamic psychotherapy continued to improve beyond the end of therapy (the so-called “sleeper effect”), so that at a 2 year follow-up they were more likely to be in a non-clinical range on measures of global functioning. An RCT study was carried out in Germany to examine the effectiveness of psychodynamic therapy with adolescents with emotional disorders (co-morbid with conduct disorders) in an inpatient setting (Salzer et al., 2014). Sixty-eight adolescents (14–19 years old) were randomized to receive inpatient psychodynamic treatment or to be in the waitlist group (Salzer et al., 2014; Cropp et al., 2019). Those who received treatment had significantly better outcomes (both at end of treatment and at 6 month follow-up) on a range of internalizing and externalizing symptoms, as well as reflective functioning, but not on psychological distress.

The largest naturalistic evaluation of psychodynamic therapies for children with emotional disorders was the Anna Freud Centre retrospective study (Fonagy and Target, 1996). The findings showed that the majority of the 299 children (85%) showed a favorable response (Target and Fonagy, 1994a). In general, those children diagnosed with emotional disorders did better than those with behavioral disorders. This finding is supported by other studies of mixed diagnostic groups, discussed elsewhere in this review, which also appeared to show that psychodynamic psychotherapy is particularly effective in reducing internalizing symptoms (Baruch, 1995; Kronmuller et al., 2005; Deakin and Nunes, 2009; Krischer et al., 2013; Ryynänen et al., 2015).

Overall, the majority of the research shows that children with emotional disorders respond well to psychodynamic therapy; indeed, this kind of therapy is often shown to be more effective for internalizing than externalizing disorders. Findings also show that young people with more severe disorders including complex comorbidities can benefit from psychodynamic therapy in an inpatient setting. Some studies demonstrate evidence of a “sleeper effect” beyond the end of treatment; this could be investigated further with more longitudinal research. Notably, the majority of the research conducted on young people with emotional disorders has focused on children of primary school age. As the following sub-sections suggest, this may be because, on reaching adolescence there is a greater likelihood that diagnosis of a specific type of emotional disorder will be made.

#### Depressive Disorders

Depression is one of the most common reasons for young people to seek mental health support in the UK. Figures suggest that 2.1% of young people aged 5–19 are diagnosed with depression, with rates of depression increasingly significantly after the age of 12 (Sadler et al., 2018). Depression is a debilitating condition with high risk of recurrence and is associated with both self-harm and suicidal ideation (Callahan et al., 2012).

Psychoanalytic understanding of depression has a long history, and there is now an extensive evidence base for the effectiveness of a range of psychodynamic treatments for depression in adults (Driessen et al., 2010; Fonagy, 2015). In the 1990s and early 2000s, both the Anna Freud Centre retrospective study (Target and Fonagy, 1994a) and the Heidelberg study (Horn et al., 2005) carried out retrospective analyses of children meeting the criteria for a depressive disorder. In the Anna Freud Centre study 75% of children with major depression showed reliable improvement and no depressive symptoms at the end of treatment, and those who had more intensive (4-5x per week) treatment had better outcomes than those who attended once-weekly therapy (Target and Fonagy, 1994a). Similar outcomes were found in the Horn et al. (2005) study.

These early naturalistic evaluations were followed by a multi-center randomized trial by Trowell et al. (2003, 2007, 2009, 2010), which compared time-limited individual psychodynamic therapy (with parallel parent work) and systems integrative family therapy (Trowell et al., 2007) for the treatment of depression in children aged 10 to 14 years. Both treatments demonstrated reductions in the levels of depression by the end of treatment, with approximately three-quarters of all young people no longer clinically depressed (Trowell et al., 2007). Additional analyses of this data set demonstrated that children in both groups also improved in terms of co-morbid conditions, family functioning, self-esteem and social adjustment (Garoff et al., 2012; Kolaitis et al., 2014). In the psychodynamic group, there were no relapses in the 6 months following the end of treatment.

Similar findings were found in a quasi-randomized study published in 2014 which reported on the treatment of depression in children from a wider age-range, between 3 and 21 years old (Weitkamp et al., 2014). At the end of therapy, there was a reduction in depressive symptoms for those who received psychodynamic therapy, with a large effect size based on child and parent-report. For children in the waiting list control group, there was also a significant reduction in depressive pathology when looking at the report of parents, but not based on child report. As with earlier studies, there were some indications that treatment outcomes were sustained over time, with over half of the children who had received psychodynamic therapy not suffering from a psychiatric disorder 1 year after the end of treatment.

Building on these earlier findings, the IMPACT study compared the effectiveness of two specialist therapies, Short-Term Psychoanalytic Psychotherapy (STPP) and Cognitive-Behavioral Therapy (CBT), with a brief psychosocial intervention (BPI), in the treatment of adolescent depression (Goodyer et al., 2011, 2016). This study, the largest and best-designed clinical trial of psychodynamic therapies for young people to date, included 465 adolescents (aged 11–17), recruited from public health services in the UK, who met criteria for moderate to severe depression. STPP was found to be equally effective as CBT and BPI both at the end of treatment, and in maintaining reduced depressive symptoms a year after the end of treatment, with 85% of young people in the STPP arm of the study no longer meeting diagnostic criteria for depression. Improvements were also observed with regard to anxiety, sleep impairment and obsessive-compulsive symptoms, as well as general psychopathology (Aitken et al., 2020; Reynolds et al., 2020). Interestingly, ending therapy prematurely was not in itself associated with poorer outcomes in the IMPACT study (O'Keeffe et al., 2019), although it appears that certain sub-groups of those who dropped out may have poorer outcomes, possibly associated with unresolved ruptures in the therapeutic alliance (O'Keeffe et al., 2020). Unlike most previous studies, all three treatments in the IMPACT study were manualised, and an assessment of treatment fidelity and differentiation confirmed that STPP was largely delivered “on model” and could be clearly differentiated from CBT and BPI (Midgley et al., 2018). The three treatments were also found to be equally cost-effective.

An interesting addition to the evidence-base for psychodynamic therapy with depressed adolescents comes with ERiCA study by Lindqvist et al. (2020), which examined the effectiveness of Internet-based psychodynamic therapy (IPDT). IPDT is a mostly self-guided treatment consisting of 8 modules delivered over 8 weeks on a secure online platform, alongside a weekly 30 min online instant-messaging chat between the young person and a therapeutic support worker. Seventy-six adolescents (aged 15–18) with unipolar depression, were randomized to either IPDT or a control condition involving online therapist support with weekly monitoring of symptoms. The study demonstrated a statistically significant weekly decrease in depressive symptoms for patients in the IPDT group compared to the control group, with these gains maintained at 6 month follow-up. Outcomes also favored IPDT compared to the control condition for all the secondary outcome measures, and the between-group effect size at the post-treatment assessment point was in favor of IPDT. The intervention is now being tested in a large-scale RCT, where IPDT will be directly compared to an internet-based CBT programme (Lindqvist et al., 2020).

Taken together, the substantial evidence-base described here supports the view that psychodynamic therapy is effective for depression in children and young people, with outcomes at least comparable to other evidence-based treatments, such as systemic family therapy or CBT. This supports the guidance of the National Institute of Clinical Health and Excellence (NICE) in the UK that STPP should be offered as one of a range of treatment options for children and young people with depression (National Institute for Clinical Excellence (NICE), 2019). There are also promising indications that novel adaptations of psychodynamic therapy, including internet-based treatment, may also be effective.

#### Self-Harm

Self-harm is common in young people, especially adolescents, and often co-occurs with a range of other difficulties, including depression, anxiety and emerging personality disorder. Two studies have specifically evaluated psychodynamic treatments for reducing self-harm. The first (Rossouw and Fonagy, 2012) compared Mentalization-Based Treatment for Adolescents (MBT-A) with Treatment as Usual (TAU), which included a range of specialist therapies usually offered in a child and adolescent mental health service. MBT-A was a year-long, manualized, psychodynamic treatment, comprising weekly individual sessions and monthly family sessions. Eighty participants were recruited into this pragmatic RCT. The study found significantly greater reductions in self-harm and risk-taking behavior for the MBT-A group, with a 44% recovery rate compared to 17% in the TAU group.

The second study to investigate treatment for reducing self-harm also evaluated a mentalization based intervention (Griffiths et al., 2019). This study was a randomized controlled feasibility trial, comparing combined MBT-A and treatment as usual (TAU) (*n* = 26), to TAU alone (*n* = 27). MBT-A was delivered to adolescents in a group format, up to 12 sessions. The findings showed that self-reported self-harm and emergency department presentation for self-harm significantly decreased over time in both groups, though there were no between group differences. Social anxiety, emotion regulation, and borderline traits also significantly decreased over time in both groups.

Overall, the findings of both these studies suggest that a contemporary psychodynamic therapy such as mentalization based treatment may be effective for treating self-harm, but further research is required, perhaps comparing treatment to a waitlist control, or to a specific alternative psychotherapy, such as CBT.

#### Anxiety Disorders

Anxiety disorders are one of the most common reasons for referral to child and adolescent mental health services. However, only a small number of studies (4) have specifically evaluated the effectiveness of psychodynamic therapy with this clinical population, with only one of these being a RCT (Salzer et al., 2018). Of these four, two focussed on anxiety disorders in general, one focused specifically on Social Anxiety Disorder (Salzer et al., 2018), and one focused on Obsessive Compulsive Disorder (Apter et al., 1984). Additionally, two papers report a re-analysis of a subset of data taken from a larger study, in one case the re-analysis focuses specifically on Separation Anxiety Disorder (Muratori et al., 2005).

A German study by Göttken et al. (2014) recruited 30 children aged 4–10 years, diagnosed with anxiety disorders. Eighteen were allocated to receive 20–25 sessions of Psychoanalytic Child Therapy (PaCT), and 12 were allocated to a waitlist control group. Based on intent-to-treat analyses, 60% of the treatment group had remitted by the end of treatment, whereas no participants in the waitlist group had remitted by the end of the waitlist. Treatment effects were maintained at 6 month follow-up according to teacher and parent reports, but child-report measures did not show a significant treatment effect at follow up.

In another study conducted in Germany, Weitkamp et al. (2018) used a quasi-experimental design to compare outcomes of a group of children and adolescents aged 4–21 years receiving psychodynamic therapy (*n* = 86), with those of a waitlist control group (*n* = 35) who received “minimal supportive treatment.” As treatments were open-ended in length, the first 25 sessions were classified as “the first treatment period,” at which point comparison was made with the waitlist control group. Overall, the findings suggest that in the first treatment period, psychoanalytic therapy had no advantage over minimal supportive treatment. However, across the whole long-term therapy period, anxiety symptoms were significantly reduced, and this remained stable at 12 months follow-up.

The best designed study of psychodynamic therapy for children with anxiety disorders was carried out by Salzer et al. (2018). This study included 107 adolescent patients, aged 14–20, diagnosed with Social Anxiety Disorder (SAD): randomized to CBT (*n* = 34), PDT (*n* = 34), or Wait List (*n* = 39). In both CBT and PDT, an identical dosage of 25 individual treatment sessions was offered (with some twice-weekly sessions at the start of treatment); therapy sessions were recorded and assessed for treatment fidelity. Both active treatments were superior to the waitlist condition with regard to reducing anxiety symptoms, with medium-to-large effects for CBT and medium effects for PDT; these effects were stable at the 12 month follow-up.

Overall, the evidence to date suggests that psychodynamic therapy, even when relatively short-term (<30 sessions) is effective in the treatment of anxiety disorders, and that these outcomes are maintained at a 6 month follow-up period. However, one quasi-experimental study seems to suggest that longer-term therapy might be required to see improvements beyond those also seen in a “minimally supportive” waitlist control. Future research could consider the relative benefits of long and short-term therapy, utilizing experimental designs with larger samples of young people, with a focus on common yet under-researched conditions such as OCD.

### Eating Disorders

The diagnostic group “feeding and eating disorders” comprises a number of related conditions, including Anorexia Nervosa and Bulimia Nervosa, which most frequently effect adolescents. One report states that 0.4% of 5–19 year-olds in the UK experience an eating disorder (Sadler et al., 2018). However, the long-term consequences of eating disorders can be severe, with studies suggesting that 20% of young people with an eating disorder may have chronic symptoms that persist into adulthood (Wonderlich et al., 2012).

In this review, five studies were identified evaluating psychodynamic therapy for eating disorders: 3 focus on Anorexia; one on Bulimia; one on eating disorders with co-occurring Addictive and/or Impulse Control Disorder; and one on children's “Feeding and Evacuation disorders.” The latter is the only study to examine a population of pre-school aged children.

Three studies have examined psychodynamic psychotherapy for the treatment for anorexia nervosa. Building on the promising findings of a small-scale study (Vilvisk and Vaglum, 1990), two studies of Adolescent Focused Psychotherapy (AFT) have been carried out, evaluating this approach in comparison to behavioral family systems therapy (Robin et al., 1995, 1999) and to Family Based Treatment (FBT, Lock et al., 2010). Both of these studies found that both treatments were similarly effective in producing full remission at the end of treatment. In Lock et al. (2010)'s study, improvement was maintained at both six- and 12-month follow-up, although levels of full remission were higher in the FBT group. A more recent study of year-long psychodynamic psychotherapy for patients diagnosed with eating disorders also found significant improvements post-therapy (Strangio et al., 2017).

Only one study has focused specifically on Bulimia Nervosa. Stefini et al. (2017) conducted an RCT comparing the effect of psychodynamic psychotherapy and cognitive behavioral therapy in a sample of 81 female adolescents with bulimia. Patients received therapy for 1 year (~60 sessions). Findings showed positive results that were broadly similar across the two treatments. A third of participants in both groups fully recovered. Overall, these findings indicate equal efficacy of both types of therapies in treating binge eating disorders.

In the only study of eating disorders in younger children, Chirico et al. (2019) investigated the efficacy of focal play therapy (FPT) for 17 children aged 2–5 experiencing “eating and evacuation” disorders. The treatment involved weekly alternate play sessions with the child and parents together, and sessions with parents only. Findings showed that the first 6 sessions were effective in promoting a positive parent-therapist alliance; however changes in parents' distress and parent-child relationship quality post-treatment did not reach statistical significance.

Overall, the evidence suggests that psychodynamic therapy can be effective in the treatment of eating disorders, with most research to date focused on anorexia and bulimia. Three RCTs have been conducted, comparing forms of psychodynamic therapy to CBT and Behavioral Family Systems Therapy. In all three trials, both treatment arms were shown to be similarly effective, suggesting that psychodynamic psychotherapy is one of a number of effective psychotherapies.

### Behavioral Disorders

Behavioral disorders (also called “externalizing” or “disruptive” disorders) are relatively common in children and young people, effecting about 4.6% of 5–19 year olds (Sadler et al., 2018), and are more common in boys than in girls (Samek and Hicks, 2014). Behavioral disorders are characterized by aggressive, inattentive, and impulsive behaviors. These disorders can have long-term negative consequences including impaired academic progress, substance use problems, and higher rates of involvement with criminal justice services in adulthood (Erskine et al., 2016).

Although disruptive disorders are a common reason for referral to child mental health services, only six studies have specifically examined the efficacy of psychodynamic psychotherapy for these children. Three of these involve a mixed population including children diagnosed with Oppositional Defiant Disorder (ODD), Disruptive Disorder, Conduct Disorder (CD) and Attention Deficit Hyperactivity Disorder (ADHD) (Eresund, 2007; Laezer, 2015; Weitkamp et al., 2017). One study focused on children and young people specifically diagnosed with CD (Edginton et al., 2018), and one focused on children diagnosed with ODD (Prout et al., 2019). One study of hyperactive children was too poorly designed to draw conclusions (Jordy and Gorodscy, 1996). In addition to these studies, two papers have reported secondary analyses of larger studies of mixed populations, with the secondary analyses focusing on outcomes for those children with a range of externalizing disorders (Fonagy and Target, 1994; Winkelmann et al., 2000).

Weitkamp et al. (2017) conducted a partly controlled, dual-perspective study, evaluating the effectiveness of psychoanalytic psychotherapy for children and young people with “severe” externalizing problems including CD, hyperkinetic disorders, and social functioning disorders. Similar to their 2018 study (reported above), the authors compared outcomes of a group of children and young people aged 4–21 years receiving psychodynamic therapy (*n* = 65), with those of a waitlist control group (*n* = 28) who received “minimal supportive treatment” after the first 25 sessions. Results showed that both groups improved with small effect sizes and no significant group differences. However, at the 1 year follow-up, significant improvements were reported in the treatment group, with higher levels of improvement were reported in patients with depressive status.

The large retrospective study from the Anna Freud Centre (Fonagy and Target, 1996) examined findings for a sub-sample of children with externalizing disorders. Results showed that overall children with a diagnosis of disruptive disorder were less responsive to treatment, and most likely to drop out of treatment (Fonagy and Target, 1994). Despite this, 46% of the sub-sample of 135 children showed improvement (69% of those who remained in treatment). Similar findings were noted in the study by Winkelmann et al. (2000), who examined the outcomes of short-term psychodynamic psychotherapy for children with behavioral disorders. The findings showed that 31% of the children in the treatment group experienced clinically significant improvement compared with 8% of those in the control group. Laezer (2015) conducted a controlled observation study involving 73 children aged 6–11, with ODD or ADHD (which DSM-5 categorizes as a neurodevelopmental disorder). One group of participants received psychoanalytic psychotherapy, whilst the other group received behavioral therapy and/or medication. Both groups experienced significantly reduced symptoms, with no significant differences between the two groups.

Given that behavioral treatments are often considered to be a first-line treatment for children with disruptive disorders, it may be important to identify specific sub-groups of children who are likely to benefit from a psychodynamic or psychoanalytic approach. Edginton et al. (2018) conducted a feasibility RCT of manualized psychoanalytical psychotherapy compared to treatment as usual for children aged 5–11 experiencing treatment-resistant CD. Thirty-two parent-child dyads participated. Though the study was not powered to evaluate outcomes, findings indicate a more promising effect on behavior problems as rated by teachers, compared to those rated by primary carers.

Overall, the studies reported here show promising findings regarding the effectiveness of psychodynamic therapy for children with externalizing disorders. However, there is some evidence that children and young people with externalizing disorders respond less well to psychodynamic therapy than those with internalizing disorders, in part because the former are more likely to drop out of treatment early. Children experiencing internalizing symptoms alongside externalizing disorders may have better outcomes. The majority of the studies that have been conducted with this group of children have small sample sizes, limiting the conclusions that can be drawn. The feasibility trial conducted by Edginton et al. (2018) suggests that larger scale studies can be conducted, indicating that RCTs should be organized in the future in order to strengthen the evidence base, comparing psychodynamic therapy to both TAU and alternative evidence-based psychotherapies.

### Children Who Have Experienced Trauma, Neglect, Abuse, or Family Conflict

One in five adults in the UK are estimated to have experienced at least one form of child abuse before the age of 16 (Office for National Statistics, 2020). Experiences of various types of abuse are even higher in clinical populations presenting to mental health services (Springer et al., 2003; Chapman et al., 2007), although exact levels of prevalence are not easy to establish. The harmful effects of maltreatment can be long-reaching and wide-ranging, which makes finding effective treatments important (Fisher, 2015).

A number of studies have investigated the outcomes of psychodynamic psychotherapy for children who have experienced trauma or early adversity: 8 have focused on children who have experienced various types of maltreatment or abuse, including children adopted or in foster care, and 3 on children exposed to parental conflict. A number of these interventions are delivered to parents rather than children, though the goal is to improve the child's well-being.

#### Children Who Have Experienced Trauma and Abuse

The Tavistock study of children in the care system (Lush et al., 1991, 1998; Boston and Lush, 1994; Boston et al., 2009) was one of the earliest studies of psychodynamic psychotherapy with children who have experienced abuse; this study gave some preliminary indication of the effectiveness of this approach. The first RCT, however, was conducted by Trowell et al. (2002), involving 71 girls (aged 6–14) who had been sexually abused. One group received focused individual psychodynamic psychotherapy for up to 30 sessions. The other group received up to 18 sessions of psycho-educational group psychotherapy. Findings showed both treatments to be effective. Individual psychoanalytic psychotherapy appeared to have a greater impact on PTSD symptoms, compared to group treatment.

Gilboa-Schechtman et al. (2010) conducted a pilot RCT for adolescents with PTSD. One group received a developmentally adapted prolonged exposure therapy for adolescents (PE-A), whilst the control group received time-limited psychodynamic therapy. Both treatments resulted in decreased PTSD symptoms and increased functioning across a range of measures. Treatment effects were maintained in both groups at follow-up.

Some studies have focused specifically on children in foster care (e.g., Clausen et al., 2012). Midgley et al. (2019) conducted a feasibility RCT with follow-up at 12 and 24 weeks post-randomization, examining the effectiveness of MBT vs. usual care (UCC) for children in foster care. Participants were 36 foster children (aged 5–16) referred to a targeted mental health service. As a feasibility pilot, the study was not powered to detect group differences in outcomes, but a preliminary analysis of outcomes indicated significant benefits for MBT compared to UCC for child-reported internalizing problems. In contrast, for the carer-reported outcome, the usual care group reported an improvement over time which was not reported in the MBT group.

Other studies have focused on children in post-adoption services. Midgley et al. (2018) conducted a naturalistic, pre-post evaluation of a short-term (six-session) mentalization-based service, “Adopting Minds”, offered to 36 adoptive families (42 adopted children). Results showed positive outcomes with a reduction in emotional and behavioral problems in the children and increased levels of self-efficacy in adoptive parents.

Polek and McCann (2020) conducted a feasibility study evaluating the effectiveness of “Adopting Together,” a time-limited psychodynamic couple-focused therapy model for adoptive couples. Fifty couples were offered therapy and outcome data were collected at intake, after 10 weeks of therapy, and after completion at 20 weeks. Although the intervention did not involve direct work with the children, results showed a reduction in parent-rated child mental health difficulties. Participants' also reported a significant reduction in depression and parenting stress, and improved relationship quality within the parenting couple.

#### Children Impacted by Parental Conflict or Domestic Violence

Research confirms that poor relationships between parents, and particularly parental conflict, can damage children's emotional well-being (Harold and Sellers, 2018). Indeed, a new condition, “child affected by parental relationship distress” (CAPRD), was introduced in the DSM-5, reflecting the impact that parental conflict, domestic violence, and acrimonious divorce/separation can have on children's mental health (Bernet et al., 2016).

Three studies published since 2017 focus on psychodynamic interventions for children affected by parental conflict or domestic violence. Of the three studies identified, one intervention was delivered to the parents (with child outcomes collected), and two interventions were delivered directly to both the child and parent together.

Pernebo et al. (2018) designed a quasi-experimental study to measure the effectiveness of two group-based interventions for children who had witnessed domestic violence between their parents. Participants were 50 children aged 4–13 years, and their mothers (in all cases, the mother was the “non-offending parent”). The treatment group (*n* = 20) received a psychotherapeutic treatment based on trauma theory, attachment theory, and psychodynamic theory within an outpatient child and adolescent mental health unit. The comparison group (*n* = 34) received a psycho-educative intervention provided at a unit offering services in the community. Children in both intervention groups experienced improvements, though symptom reduction was larger in the psychotherapeutic intervention, and children with initially high levels of trauma symptoms benefited the most. However, most mothers reported child trauma symptoms at clinical levels at the end of treatment.

Bernstein et al. (2019) conducted a RCT with a group of 113 mothers who had experienced interpersonal violence, and their young children (aged 2–6). The authors tested whether Child-Parent Psychotherapy, a treatment based on psychoanalytic principles, can change biases in mothers' perceptions of their child's facial expressions, and consequently reduce child symptomology. In the study, 80 mother-child dyads received CPP, and 33 received supportive case management with individual psychotherapy for the mother and/or child. Results showed that mothers who participated in CPP showed significant reductions in bias toward fear (but not anger) from post-treatment to baseline, whereas mothers in the treatment-as-usual group showed no significant change.

Hertzmann et al. (2016, 2017) designed a MBT intervention for parental couples experiencing high levels of conflict post separation/divorce (MBT-PT). This was a pilot feasibility study, with 15 pairs of co-parents randomly allocated to either MBT-PT (*n* = 15), which parents attend together as a couple over 6–12 sessions, or to Separated Parents Group (PG), a psycho-educational intervention for separated parents. Results showed that parents in both interventions reported significantly less expression of anger toward each other over the period of the study. This may reflect parents' improved capacity to mentalize and control their own feelings toward the co-parent, resulting in reduced expressed anger or conflict that might impact the child. However, there was no significant difference between the two interventions.

Overall, these studies suggest promising findings for the use of psychodynamic treatment with children who have experienced parental conflict and/or trauma, including those who are in foster care or who have been adopted. Results show potential for increased well-being for children, and decreased stress for their carers. However, research is still limited and most of the studies conducted in this area are with small samples in naturalistic studies. Future research should involve larger samples using an experimental design.

### Emerging Personality Disorders (PD)

Although the concept of personality disorder (PD) is well-established in relation to adults, there is on-going debate about whether the term can appropriately be used in relation to adolescents (Lenkiewicz et al., 2015), and hesitance among some professionals in making this diagnosis in young people (Hauber et al., 2017). There is, however, increasing evidence to suggest that emerging PD is a meaningful construct when thinking about adolescent psychopathology (Paris, 2013), and this is reflected in the research on emerging personality patterns in adolescence set out in the revised edition of the Psychodynamic Diagnostic Manual (Malone and Malberg, 2017).

In our review, we found 8 studies investigating psychodynamic psychotherapy in the treatment of young people with PD, with the number of studies clearly increasing over time. A significant proportion of these studies involved adapted versions of MBT, which it is not surprising given that this model of psychodynamic therapy it is established as an evidence-based treatment for Borderline Personality Disorder in adults (BPD; Bateman and Fonagy, 2010; Storebø et al., 2020). Of the eight studies identified, six focused specifically on Borderline Personality Disorder (BPD), one on Avoidant Personality Disorder (APD), and one included patients with various PDs or traits. All studies involved adolescents aged 14 and over.

Chanen et al. (2008) conducted an RCT evaluating the effectiveness of cognitive analytic therapy (CAT) vs. usual clinical care for outpatient adolescents aged 15–18 who fulfilled two out of nine of the DSM-IV criteria for BPD. Overall, the two interventions were found to be equally effective. Both treatment groups demonstrated significant improvements which were maintained at follow up, including substantial reduction over time in the chances of parasuicidal behavior.

Naturalistic evaluations of psychodynamic treatment of BPD have shown promising results. Salzer et al. (2014) conducted on an observational study assessing the effectiveness of psychodynamic psychotherapy with 28 adolescents with BPD. Pre-post analyses showed that 39.3% of the patients were remitted by the end of treatment, in addition to significant improvements on a range of other measures. Likewise, Schenk et al. (2019) conducted a small exploratory study of psychodynamic therapy, involving 10 adolescents (aged 14–18) with identity diffusion and BPD symptoms. Results showed a significant reduction in psychopathology and an improvement in psychosocial functioning over time. A study by Sugar and Berkovitz (2011a,b) gives some indication that improvements can be maintained through to adulthood, although the study was unsystematic and had a very small sample.

Of the 2 MBT studies for BPD, one was a naturalistic pre-post evaluation, the other was a RCT. Bo et al. (2017) evaluated the effectiveness of a group-based MBT (MBT-G) for 34 female adolescents (aged 15–18). Twenty-five adolescents with BPD completed the study, of which the majority (*n* = 23) displayed significant improvement regarding borderline symptoms, depression, self-harm, peer-attachment, parent-attachment, mentalizing, and general psychopathology. Building on this, Beck et al. (2020) conducted an RCT evaluating the effectiveness of a group-based MBT (MBT-G) vs. treatment as usual (TAU) for adolescents aged between 14 and 17 with BPD. In both treatment arms, there was a statistically significant improvement, although it was considered clinically insignificant. No significant between-group differences were found in outcomes. A 3 and 12 month follow-up showed that both groups demonstrated improvement in the majority of clinical and social outcomes at both follow-up points (Jørgensen et al., 2020).

The effectiveness of MBT has also been evaluated for other PDs. Bo et al. (2019) reported on the effectiveness of an adaptation of MBT for 8 adolescents (aged 14–18) with Avoidant Personality Disorders (APD) (MBT-AA; Bo et al., 2019). Findings showed a significant change in avoidant personality pathology from baseline to end of treatment. At the end of treatment all patients scored below the cut-off point for APD. Furthermore, there were significant improvements in internalizing pathology, mentalizing, and peer- and parent attachment, but not for externalizing psychopathology. Similar results were found by Hauber et al. (2017), who examined the effectiveness of an intensive MBT with a psychodynamic group psychotherapy approach involving partial hospitalization, in which adolescents showed a significant reduction in personality disorder traits and symptoms by the end of treatment.

Overall, these studies provide some preliminary support for the use of psychodynamic psychotherapy in the treatment of PDs, especially BPD, in adolescence. In particular, the evidence for various adaptations of MBT are promising and suggest that this model of psychodynamic treatment for adolescents with PDs may be particularly effective. However, only two of the six studies were RCTs; the others were all naturalistic pre-post studies, mostly with small sample sizes, and lacking long-term follow-ups. Given these methodological limitations, further research is needed to draw more robust conclusions about the effectiveness of psychodynamic treatments for PD in young people. Such research is especially important given the robust evidence-base in adults, and the costs to individuals, services and society of PDs.

### Children With Neuro-Developmental Disorders

Neuro-developmental disorders—sometimes referred to as learning disorders/disabilities—comprise a range of diagnoses (Reiss, 2009). Some classification systems also include Attention Deficit / Hyperactivity Disorder (ADHD) in this category, although for the purposes of this review studies of ADHD have been reviewed in the section on “Behavioral Disorders.”

Children diagnosed with neuro-developmental disorders may experience limitations in core functional domains (e.g., motor, communication, social, academic) resulting from abnormal development of the nervous system (Reiss, 2009). Although these disorders are not usually considered “mental illness,” but developmental disorders; they overlap with and are risk factor for mental illness (Eapen, 2014). Therefore, the emotional or behavioral issues that are often experienced alongside developmental disorders are sometimes treated with psychotherapy interventions, delivered to the child and/or caregiver.

#### Children With Specific Learning Difficulties

Just two studies examined therapy for children experiencing learning difficulties. A study by Heinicke and Ramsay-Klee (1986) looked a sample of 12 boys aged 7–10 years, referred with reading difficulties and associated “emotional disturbance.” The children received group-based psychoanalytic psychotherapy over a period of 2 years. All participants improved with treatment, particularly with regard to self-esteem, flexible adaptation, capacity for forming and maintaining relationships, frustration tolerance, and ability to work.

Zelmann et al. (1985) also found psychoanalytic treatment to be effective in increasing the IQ of young children (mean age: 3 years 8 months) experiencing developmental language delay. However, the sample of this study was small and therefore the findings should be treated with caution.

Although these two studies showed positive improvements for participants in terms of increased IQ and greater well-being, it is not possible to draw general conclusions from this limited research. Larger, controlled studies are required.

#### Autism Spectrum Disorder

Autism Spectrum Disorder (ASD) is a heterogeneous neurodevelopmental disorder characterized by deficits in social interaction and social functioning, and by certain repetitive behaviors and restricted interests. To date there has been only one empirical study of the effectiveness of this therapeutic approach for children with ASD. This quasi-experimental study focussed on children with ASD and their families (Enav et al., 2019). This study sought to improve parents' capacities to mentalize and regulate their emotions, such that they are better able to manage their child's behavior. In this sample, 64 parents of children with ASD (child aged 3–18) were allocated to a 4 week, group mentalization-based treatment, or to a delayed-treatment control. The findings showed that, compared to delayed treatment group, parents in the mentalization-based group had increases in reflective functioning and in the belief that emotions can change. Moreover, they reported decreased behavioral and emotional symptoms in their children, and greater parental self-efficacy.

Overall, there is limited research focusing on psychodynamic approaches to neuro-developmental disorders, with no RCTs to date. Future research should use an RCT design with larger samples and robust assessments of child/parent outcomes.

### Children With a Physical Illness

A small number of studies have examined the impact of psychodynamic therapy on children and young people with a physical illness, especially in situations with psychological factors may impact on a child's capacity to manage their physical health condition.

Moran et al. undertook a series of high quality studies examining the use of intensive psychoanalytic psychotherapy (3–5 sessions per week for a mean duration of 15 weeks) as a means of helping young people with poorly controlled diabetes (Moran and Fonagy, 1987; Fonagy and Moran, 1990; Moran et al., 1991). Treatment was compared to a control group of adolescents who received routine psychological input only. Findings showed that young people in the treatment group experienced a significant improvement in diabetic control compared to the control group. This improvement was maintained at 1 year follow-up (Moran et al., 1991).

The only other study focussing on physical health was a pilot RCT, investigating the treatment of idiopathic headache (Balottin et al., 2014). In this study, brief psychodynamic psychotherapy was found to be significantly more effective than care as usual in reducing headache frequency, intensity, and duration.

Overall, there is a limited amount of research evaluating the use of psychodynamic or psychoanalytic therapy for children with physical health conditions, though the research that has been done is of good quality, mostly using randomized or quasi-randomized designs.

### Practice-Based Evidence for Psychodynamic Therapy With Mixed Groups of Children

When comparing the research in child and adolescent psychodynamic therapy identified in more recent reviews with to earlier ones, it is noticeable that there has been a change in the direction and focus of research over time. Studies are increasingly experimental in design, focusing on a particular diagnostic or clinical group, rather that analyzing data routinely collected in a naturalistic setting with children presenting with a mix of clinical difficulties.

Whilst this perhaps reflects growing recognition of the need to rigorously assess the efficacy of psychodynamic therapy by both researchers and funders, it is important not to overlook the value of naturalistic studies conducted in a real-world setting. “Practice-based evidence” involves monitoring routine clinical practice, and observing what therapists actually do in their regular everyday activity as a means of studying what works (Manning, 2010). Whilst experimental designs may provide a more rigorous form of evaluation and help to establish the efficacy of a particular type of therapy, they do not always help us to understand what the effectiveness of routine psychodynamic therapy may be. Arguably, the findings of these naturalistic, effectiveness studies are more reflective of the kinds of outcomes experienced by children in “real world” healthcare settings (i.e., they have good “external validity”), and therefore have clear implications for usual clinical practice.

In this review, we identified 29 studies of mixed diagnostic groups, nearly all of which were conducted in naturalistic settings. In what follows, we describe some of the larger and better-designed studies.

The majority of the studies of mixed populations focused on the treatment of children (aged 3–12). For example, Edlund et al. (2014) conducted a naturalistic study, with a relatively large sample of 207 participants aged 4–12 years. Results showed that psychodynamic psychotherapy was associated with a significant improvement in functioning, with a large effect size. In a comparable study, conducted in Brazil, Deakin and Nunes (2009) looked at the effectiveness of child psychoanalytic psychotherapy for a sample of 23 children aged 6 to 11 years, experiencing a range of psychological disorders. Findings showed that children who received treatment experienced a significant reduction in total internalizing and externalizing difficulties after 12 months of treatment, in addition to improved interpersonal relationships and affect regulation. Treatment was most effective for girls with internalizing problems. Similar results have been found by studies in other countries. In an analysis of 89 children from Turkey aged 4–10 years old, experiencing a range of problems, Halfon et al. (2019a,b) found that 54% of the children showed reliable improvement in externalizing and internalizing problems at the end of treatment.

There is a considerable amount of practice-based evidence related to the psychodynamic treatment of adolescents. For example, in a community-based study of psychodynamic treatment for adolescents and young adults presenting with multiple difficulties, findings show that measurable change took place during the course of therapy in all domains of functioning (Baruch, 1995). However, “externalizing” problems were more difficult to treat than “internalizing” problems, although those with externalizing problems did better if they also presented with emotional problems or if the individual was in more intensive treatment. The sample has been followed up at a number of points (Baruch et al., 1998; Baruch and Fearon, 2002; Baruch and Vrouva, 2010).

Tonge et al. (2009) conducted a longitudinal naturalistic study of psychoanalytic psychotherapy for adolescents with serious mental illness. The study compared outcomes for 40 adolescents who received psychoanalytic psychotherapy once or twice weekly, with 40 adolescents who received treatment as usual (TAU). The findings showed those treated with psychodynamic psychotherapy experienced a greater reduction in both mental health symptoms and social difficulties compared with those in the TAU group; however the greater effectiveness of the psychodynamic treatment depended on initial level of symptomatology, with a “floor effect” identified.

Two publications have resulted from a naturalistic study of adolescents receiving psychodynamic psychotherapy in outpatient clinics in Israel. The treatment group comprised 72 adolescents (aged 15–18), and the comparison group was a non-clinical community control. Findings showed that those in the treatment group became less rigid in their interpersonal patterns, developed more adaptive internal representations of relationships with parents, and experienced significant symptom reduction. No such changes were observed in the community sample (Atzil Slonim et al., 2011, 2013). Similar findings were reported by Tishby et al. (2007), in a small study of changes in interpersonal conflicts among adolescents during psychodynamic psychotherapy.

Overall, the studies of psychodynamic therapy for children and adolescents in naturalistic settings show encouraging findings. Although such evidence does not carry the same weight in most guidelines on evidence-based practice, these naturalistic studies can be seen as offering a “bottom-up” model, whereby routine data is gathered at a service-level, with the possibility that findings can gradually be accumulated across services. Such an approach is in line with the increasing emphasis on models of quality improvement within mental health services (Ross and Naylor, 2017), and may give a more realistic sense of how psychodynamic therapies impact on the lives of children and families referred to mental health services.

## Discussion

The aim of this review was to provide a narrative synthesis of the evidence base with regard to psychodynamic therapy with children and adolescents. In order to do this, an updated search covering research published between between January 2017 and May 2020 was conducted, and the findings from this search were then synthesized with those reported in two earlier reviews (Midgley and Kennedy, 2011; Midgley et al., 2017).

This updated search identified 37 papers published between January 2017 and May 2020, reporting on 28 distinct studies. These were combined with the findings of the previous reviews, to total 123 papers, comprising 82 distinct studies.

Overall, both the quality and quantity of research in this field has increased over time. For example, the proportion of studies using an experimental and quasi-experimental design has grown with each update of the review. This is especially important given that many clinical guidelines only draw on evidence from studies with such designs. Nevertheless, the majority of studies in this review were conducted in naturalistic settings using clinically referred rather than recruited samples. Many used an observational design, though some included matched community or TAU control groups. Whilst the findings of these studies cannot be considered as “rigorous” as those of experimental studies, such studies may be more representative of a “real-world” context, where treatments are not often delivered according to a specific manual, treatment length is not predetermined, and patients often present with a mixed picture of mental health issues. The large number of studies in this area means that there can be greater confidence that any outcomes identified in more controlled settings can be replicated in routine clinical practice.

The research synthesized in this study makes it possible to draw some tentative indications about who is likely to benefit most (or least) from psychodynamic child psychotherapy. Based on the studies reviewed here, the following initial conclusions can be drawn:

- There have been a relatively large number of studies evaluating the outcome of psychodynamic therapies for children with emotional disorders: 21 studies, of which 12 are RCTs. Taken together, these studies indicate that emotional disorders respond well to psychodynamic therapy; with a number of studies suggesting that psychodynamic treatment is more effective for internalizing than externalizing symptoms, and that younger children are likely to show a larger treatment response.
- Within the emotional disorders category, the quality of research has been particularly high for the treatment of depression, where 3 RCTs have been conducted, including the largest study to date to include a psychodynamic treatment arm either in children or young people, the IMPACT study (Goodyer et al., 2016). Taken together, these studies indicate that psychodynamic psychotherapy has comparable outcomes to other psychological treatments such as CBT or systemic family therapy, and that it can result in good outcomes across a range of domains, with those outcomes maintained beyond the end of treatment.
- The comparative effectiveness of psychodynamic therapies also seems to be demonstrated for other disorders, such as bulimia nervosa and anorexia nervosa. Two RCTs focused on anorexia and one focused on bulimia found psychodynamic treatment to be equally effective to an alternative treatment.
- The 2017 review found no sufficiently high-quality studies in samples of children and adolescents with anxiety disorders, disruptive behavior problems, or personality disorders. Whilst there are still very few RCTs evaluating the effectiveness of psychodynamic therapies in the treatment of disruptive behavior problems in children and young people, the evidence base for anxiety and personality disorders has grown in recent years. There are now 3 RCTs focused on anxiety disorders and 2 on emerging personality disorders, with several observational studies of the psychodynamic treatment of BPD published in the last 3 years.
- For the treatment of anxiety disorders, a number of studies have found psychodynamic treatment to be effective. The best designed study of psychodynamic therapy for children with anxiety disorders was an RCT carried out by Salzer et al. (2018), which showed both active treatments were superior to a waitlist condition, with medium-to-large effects for CBT and medium effects for PDT. Overall, the evidence to date suggests that psychodynamic therapy, even when relatively short-term (<30 sessions) is effective in the treatment of anxiety disorders, and that these outcomes have been maintained at a 6 month follow-up period.
- There is evidence to suggest that a contemporary psychodynamic therapy such as mentalization based treatment may be effective for treating self-harm in adolescents. Two RCTs have been conducted to date, and both demonstrated that a mentalization based intervention was equally or more effective than TAU for the treatment of self-harm.
- Comparatively, the psychodynamic treatment of externalizing disorders has received less research attention, and this may partly be because the evidence-base for a range of parenting interventions in this area is well-established (Fisher, 2015). There have been only 6 studies of psychodynamic therapies for this group of children, and only one of these was an RCT. However, despite the accepted wisdom that non-behavioral therapies are less effective for disruptive disorders, these studies show promising findings, particularly when the child also presents with some emotional difficulties. Research suggests that children with disruptive disorders may be difficult to engage, but those who remain in treatment can see significant symptom reduction. It may be, as with the feasibility study conducted by Edginton et al. (2018), that future studies of psychodynamic therapy should focus especially on those children with disruptive disorders who have not been responsive to a first-line treatment, including parenting interventions.
- Some areas have received growing research interest in recent years, with more studies identified in more recent reviews. Emerging personality disorders have been examined in 8 studies, of which 2 are RCTs. Five of these 8 studies have been published since 2017. The two RCTs of BPD both showed the psychodynamic treatment to be equally effective to the control condition: cognitive analytic therapy (Chanen et al., 2008) and group-based MBT (Bo et al., 2017). Given the high personal and social costs of personality disorders across the lifespan, and the evidence of the effectiveness of psychodynamic therapies for adults with personality disorders (Storebø et al., 2020), this may be an area where psychodynamic therapies have an especially important role to play.
- Similarly, in recent years more studies have focused on children impacted by parental conflict or domestic violence—this review found three studies, all published since 2017, of which two were RCTs. These three studies were designed quite differently, such that it is difficult to draw together their findings. However, the study by Pernebo et al. (2019) suggests that children experiencing trauma symptoms are particularly able to benefit from group psychodynamic therapy, suggesting a promising area for future research with children impacted by parental conflict.
- Eight studies, including 3 RCTs, have evaluated the effectiveness of psychodynamic therapies with children who had experience trauma, including children in foster care and post-adoption. These findings are promising and show that psychodynamic therapy is as effective as alternative treatments (Trowell et al., 2002; Gilboa-Schechtman et al., 2010). Recent reviews of the work of psychodynamic child psychotherapists have highlighted the wide range of settings in which psychodynamic therapists work with children who have experienced maltreatment, especially those children who have been adopted or who are in care (Robinson et al., 2017, 2019, 2020). Therefore, there is an urgent need to build on the preliminary research in this area, with larger and better-designed studies.
- We identified only 2 studies examining the effectiveness of psychodynamic therapy for physical illness, though these are both well-designed. Moran and Fonagy (1987), Fonagy and Moran (1990), Moran et al. (1991) show psychodynamic therapy to be effective in the treatment of adolescents with poorly controlled diabetes. There is also evidence from a pilot RCT that psychodynamic therapy can reduce symptom severity for young people experiencing idiopathic headache (Balottin et al., 2014). These findings suggest that further research should consider psychodynamic treatments for certain physical conditions, where symptoms or treatment adherence may have an important psychological component that could be treated with psychotherapy.
- There are a number of areas where very little research has been carried out evaluating the effectiveness of psychodynamic therapies. This includes research into the treatment of children and young people with autistic spectrum disorder, OCD and the range of eating disorders. If psychodynamic therapy is to be offered to children with these clinical presentations, it is vital that more outcome research is carried out.

Although this summary indicates that we are now in a position to draw some tentative conclusions, caution is needed. The number of clinical trials evaluating psychodynamic therapies for children and young people remains very small when compared to studies of psychopharmacological interventions, or even other psychosocial treatments for children and young people, such as CBT. For example, in a systematic review of studies examining the effectiveness of CBT with children and adolescents, Oud et al. (2019) identified 31 RCTs focused on depression alone, this compares to 3 RCTs of psychodynamic therapy as a treatment for adolescent depression identified in this review. The numbers are also small compared to the research focused on psychodynamic therapy with adults, where one review has indicated that over 250 RCTs have been published to date (Lilliengren, 2017).

Of all the obstacles to further research, perhaps the lack of funding opportunities is the single biggest obstacle to further research being carried out. A report by MQ in 2017 noted that mental health research is chronically under-funded compared to physical health, but that even within mental health research, only 3.9% of funding goes toward prevention of mental illness, 5.5% toward the development of new treatments, and 18.3% to the evaluation of treatments. The report also notes that “only 26% of money spent on mental health research goes toward projects on children and young people” (MQ, 2017, p. 3). Without greater priority being given to the study of mental health interventions for children and young people, especially those evaluating treatments models beyond CBT, there is little chance that commissioners or families will be able to draw conclusions about effective therapies based on high-quality science.

The current review also suffers from a number of limitations. First, the data extraction and quality assessment process was carried out by different groups at each stage of carrying out this review (2011, 2017, and 2020), which means that there may not have been complete consistency in how this was done. Second, because of significant variation in study reporting, it was not possible to provide consistent reporting of the key study components from each study, such as how study populations were identified. Likewise, the great variation in study design—including outcome measures and methods of data analysis—meant that no meta-analysis of the data was carried out. Additionally, research examining the process of therapy (e.g., Fisher et al., 2016; Calderon et al., 2019; and for a review, Kennedy and Midgley, 2007), or qualitative studies examining the experience of psychodynamic child and adolescent psychotherapy (e.g., Løvgren et al., 2019; Marotti et al., 2020), were both beyond the scope of this report. Nor did this review include studies evaluating the effectiveness of psychodynamic therapy with parents and infants—an area where child psychotherapy has played a significant role for a number of years. Other reviews have covered this important area (e.g., Sleed and Bland, 2007; Barlow et al., 2016), but this absence means that there is a gap in the presentation of the evidence-base for psychodynamic child and adolescent psychotherapy across the whole age range.

## Conclusion

It has been reported that 75% of mental illnesses start before a child reaches their 18th birthday, while 50% of mental health problems in adult life (excluding dementia) first appear before the age of 15 (MQ, 2017). These widely quoted figures highlight the urgent need for “evidence based” interventions that limit the impact of mental health problems that may persist into adulthood, at considerable individual, social, and economic cost. This review aimed to bring together the research that has evaluated psychodynamic therapies for children and young people, to ensure that current and future decision-making in child mental health settings is informed by the best available evidence. Although the number of studies is still very small compared to other treatment modalities, there is now a growing evidence-base that suggests that psychodynamic therapies can be effective for children and young people presenting with a wide range of clinical issues.

It is clearly important to be able to systematically review the evidence-base for psychodynamic therapies with children and young people. But going forward, there is a need to balance this demand with a greater focus on practice-based evidence, including large-scale routine outcome monitoring and the emerging field of practice-research networks (Barkham et al., 2010). There is also an increasing need to pay attention to the findings of qualitative research, including studies of client experience and service-user preferences (Midgley et al., 2014). Such research can help to identify helpful and unhelpful aspects of therapy and puts the needs and experiences of children, young people and families at the heart of evidence-based practice. By widening what “counts” as credible evidence and by broadening the kind of questions we ask about that evidence, as well as promoting more interdisciplinary studies, research can truly help ensure patient choice, and to enable provision of diverse range of effective treatments, with service user experience at the heart of all decision making.

## Data Availability Statement

The original contributions presented in the study are included in the article/Supplementary Material, further inquiries can be directed to the corresponding author/s.

## Author Contributions

RM, AC, and PB were involved in the initial search and subsequent screening and assessment of included papers, under the supervision of NM. All authors were involved in the conceptualization and writing of the manuscript.

## Conflict of Interest

NM is a child and adolescent psychotherapist, and a member of the Association of Child Psychotherapists in the UK. The remaining authors declare that the research was conducted in the absence of any commercial or financial relationships that could be construed as a potential conflict of interest.

## Footnotes

^1^For the purposes of this review, the terms “child psychotherapy” and “psychodynamic child psychotherapy” will be used generically to cover psychoanalytic and psychodynamic psychotherapy with children and adolescents. It is recognized that these terms cover a broad range of approaches, which to varying degrees draw on various disciplines, including different schools of psychoanalysis, as well as developmental psychology, attachment theory, neuroscience etc. Where individual studies describe the model of therapy being evaluated, e.g., “psychoanalytic therapy,” we will follow their usage.
