# Supplementary material for: The Evidence-Base for Psychodynamic Psychotherapy With Children and Adolescents: A Narrative Synthesis
Source: Front Psychol. 2021 Apr 27;12:662671. doi: 10.3389/fpsyg.2021.662671 (PMC8110733; doi:10.3389/fpsyg.2021.662671)
Supplement: Supplementary file 1 [file Table_1.docx]

**Table 1. Search Strategy**

| **Search Terms** | **Databases Searched (January 2017 – May 2020)** |
| --- | --- |
| (child* OR teenage* OR adolescent* OR * young person* OR * young people)  AND  (psychoanaly* OR psychodynamic* OR psychotherapy*)  AND  (therap* OR intervention* OR treatment*)  AND  (efficacy* OR effective* OR outcome* OR trial* OR experiment* OR empirical* OR investigate* OR outcome*OR finding* OR result* OR measur* OR evaluat*) | PsycInfo  EMBASE  Scopus  Web of Science  CINAHL  PubMed  Medline  The Cochrane Library |

**Table 2. Data Extraction for Studies 2017-2020**

| **Author/ Date** | **Design** | **Location** | **Presenting Problem** | **Age of Participants** | **Sample Size** | **Intervention** | **Control Group / Comparison Treatment** | **Outcome Measure** |
| --- | --- | --- | --- | --- | --- | --- | --- | --- |
| Beck, et al. (2020);  Jørgensen et al. (2020) | RCT | Denmark | BPD | 14-16 | 112 | Mentalization-Based Treatment (Group) | TAU | Borderline Personality Features Scale for Children (BPFS-C); Beck’s Depression Inventory for Youth (BDI-Y); Risk-Taking and Self-harm Inventory for Adolescents (RTSHI-A); Youth Self-Report (YSR), Borderline Personality Features Scale-Parent (BPFS-P); Child Behavior Checklist (CBCL); the Zanarini Rating Scale for Borderline Personality Disorder (ZAN-BPD); the Children’s Global Assessment Scale (CGAS). |
| Bernstein, Timmons, & Lieberman (2019) | RCT | USA | Children exposed to Parental Violence | 2-5 | 113 mother-child dyads | Child-Parent Psychotherapy | Monthly case management / individual treatment in the community | The IFEEL Picture System (IFP), Clinician-Administered PTSD Scale, Child Behavior Checklist (CBCL). |
| Bo et al. (2017) | Observational without control | Denmark | BPD | 15-18 | 34 | Mentalization-Based Treatment (Group) | none | Borderline Personality Features Scale for Children (BPFS-C); The Youth Self-Report (YSR); Beck Depression Inventory for Youth (BDI-Y); Risk-Taking and Self-Harm Inventory for Adolescents (RTSHI-A); Inventory of Parent and Peer Attachment—Revised (IPPA-R); Reflective Function Questionnaire for Youth (RFQ-Y) |
| Bo, Bateman, & Kongerslev (2019) | Observational without control | Denmark | Avoidant Personality Disorder | 14-18 | 8 | Mentalization-Based Treatment | none | Millon adolescent clinical inventory (MACI); Youth self-report (YSR); Child behavior checklist (CBCL); Inventory of parent and peer attachment – revised (IPPA-R); Reflective Functioning Questionnaire for Youth (RFQY) |
| Chirico et al. (2019) | Observational without Control | Italy | Eating and evacuation disorders | 2-5 | 17 couples | Focal Play Therapy | none | The Working Alliance Inventory-Short Form (WAI-S); the System for Observing Family Therapy Alliances-Self report; the Parenting Stress Index-Short Form. Emotional Availability Scales |
| Cropp et al. (2019) | RCT | Germany | Comorbid disorders of conduct and emotions | 15-19 | 38 | Psychodynamic psychotherapy | Waitlist | The Reflective Functioning Scale (RFS); Symptom Check List 90-R; Global Assessment of Functioning Scale. |
| Edginton et al. (2018) | RCT | UK | Conduct disorders | 5-11 | 32 parent-child dyads | Psychoanalytic child psychotherapy | TAU | Child Behavior Checklist (CBCL and TRF); Parental Reflective Functioning Questionnaire (PRFQ); General Health Questionnaire 12 (GHQ-12); Parenting Stress Index (PSI); Beck Depression Inventory (BDI); EuroQol 5 Dimension (EQ-5D); EuroQol 5 Dimension Youth (EQ-5D-Y) |
| Enav et al. (2019) | Quasi-experimental | USA | ASD | 3-18 | 68 parents | Mentalization-Based Treatment | Waitlist | Parent Development Interview (PDI); The Emotion Regulation Questionnaire (ERQ); The Child Behavior Checklist (CBCL); the Aberrant Behavior Checklist (ABC); the Parenting Sense of Competence Scale (PSOC) |
| Gatta et al. (2019) | Observational without control | Italy | Mixed Diagnoses | 6-18 | 57 families (each including one minor, 2 parents) | Short-Term Psychodynamic Psychotherapy (STPP) | none | The Child Behaviour Checklist (CBCL); the Youth Self-Report (YSR); Family Empowerment Scale (FES) |
| Aitken et al. (2020); Davies et al. (2020); O’Keefe et al. (2019); O’Keefe, Martin & Midgley (2020); Reynolds et al. (2020); Goodyer et al. (2017) | RCT | UK | Major Depressive Disorder | 11-17 | 465 | Short-Term Psychoanalytic Psychotherapy (STPP) | Two comparison therapies: 1) Brief Psychosocial Intervention (BPI). 2) Cognitive-Behavioural Therapy (CBT). | The Mood and Feelings Questionnaire (MFQ); Health of the Nation Outcome Scale for Children and Adolescents (HoNOSCA); the Revised Children’s Manifest Anxiety Scale (RCMAS); the revised Leyton Obsessional Inventory (LOI); the Kiddie-SADS28. O’Keefe et al (2020) also used: the Rupture Resolution Rating System (3RS) and Working Alliance Inventory (WAI-O) observer version. |
| Griffiths et al. (2019) | RCT | UK | Self-Harm | 12-18 | 53 | Mentalization-Based Treatment (Group) | TAU | The Risk-Taking and Self-Harm Inventory for Adolescents (RTSHI); the Revised Child Anxiety and Depression Scale (RCADS); Reflective Functioning Questionnaire for Youths (RFQ-Y); Difficulties in Emotion Regulation Scale (DERS); Interpersonal Sensitivity Measure (ISM); short version of the Borderline Personality Features Scale for Children (BPFSC); short version of the Experiences in Close Relationships Scale–Revised Child version (ECRS-RC) |
| Halfon & Bulut (2017); Halfon, Cavdar, & Yilmaz (2019); Halfon, Ozsoy, & Cavdar (2019) | Observational without control | Turkey | Mixed Diagnoses | 4-10 | 89 | Psychodynamic Play Therapy | none | The Child Behavior Checklist (CBCL); the Children's Global Assessment Scale (GCAS); The HoNOSCA; the Children's Play Therapy Instrument (CPTI); the Child Psychotherapy Q-Set (CPQ); Emotional Regulation Checklist (ERC); The Therapy Process Observational Coding System (TPOCS-A) |
| Hauber, Boon, & Vermeiren (2017) | Observational without control | Netherlands | Personality Disorder | 15-22 | 63 | Mentalization-Based Treatment (Group) | none | Dutch Questionnaire for Personality Characteristics (DQPC); Symptom Checklist 90; Structured Clinical Interview for DSM Personality Disorders (SCID-PD) |
| Hertzmann et al. (2017) | RCT - feasibility | UK | Child exposed to Parental Conflict | Not Specified | 15 parental couples | ‘Parenting Together' (Mentalization-Based Group Treatment) | A psycho-educational intervention for separated parents | The Stait-Trait Anger Expression Inventory-2 (STAEI-2); the Parental Reflective Function Questionnaire (PRFQ); the Perceived Stress Scale (PSS); the Patient Health Questionnaire (PHQ-9); the Parenting Alliance Measure; the Relationship Attribution Measure (RAD); the Strengths and Difficulties Questionnaire (SDQ, parent report); the Security in the Marital Subsystem–Parent Report (SIMSPR) |
| Krischer et al. (2020) | Quasi-experimental | Germany | Mixed Diagnoses | 4-17 | 83 | Psychodynamic psychotherapy | Waiting List | Child Behaviour Checklist (CBCL); Youth Self-Report (YSR); QoL Inventory for Adolescents |
| Levy (2017) | Observational without control | USA | At risk of mental health difficulties | 3.5-6 | 11 | Relationships for Growth and Learning (RfGL) Peer Play Psychotherapy | none | The Coding Interactive Behavior (CIB) Rating Scale; the Penn Interactive Peer Play Scale. |
| Lindqvist et al. (2020) | RCT | Sweden | Major Depressive Disorder | 15-18 | 76 | Internet-based psychodynamic therapy (I-PDT) | Online Therapist Support | The QIDS-A17-SR; the Generalized Anxiety Disorder 7-item scale (GAD-7); the Montgomery Åsberg Depression Rating Scale–self-rated (MADRS-S); the Self-Compassion Scale (SCS); the Difficulties in Emotion Regulation Scale (DERS) |
| Midgley et al. (2018) | Observational without control | UK | Adopted children | 2-17 | 36 families, including 42 children | Adopting Minds (Mentalization-Based Family Treatment) | none | Brief Assessment Checklist (BAC), Brief Parental Self Efficacy Scale (BPSES); Experience of service questionnaire (ESQ) |
| Midgley et al. (2019) | RCT | UK | Children in Foster Care | 5-16 | 36 | Mentalization-Based Treatment (Family) | TAU | Strengths and Difficulties Questionnaire (SDQ); Brief Assessment Checklist (BAC); Revised Children's Anxiety and Depression Scale (RCADS); Parenting Stress Index (PSI) -short form; Parenting Efficacy Scale (PES); Parenting Scale; Goal Based Outcomes (GBOM). |
| Schenk et al. (2019) | Observational without control | Switzerland | BPD and Identity Diffusion | 4-18 | 10 | Adolescent Identity Treatment (AIT) | none | The Children’s Global Assessment Scale (CGAS); Youth Outcome Questionnaire Self-Report (Y-OQ) |
| Pernebo, Fridell, & Almqvist (2018) | Quasi-experimental | Sweden | Children exposed to inter-parental violence | Apr-13 | 50 children and 50 mothers | Trauma-focused time-limited psychodynamic psychotherapy (group) | Community-based psychoeducative intervention | The revised Conflict Tactics Scale (CTS2); the Strength and Difficulties Questionnaire (SDQ); the Trauma Symptom Checklist (TSC) for Young Children; the Emotion Questionnaire for Parents (EQ-P), the Brief Symptom Inventory (BSI); the Impact of Event Scale–Revised (IES-R) |
| Polek & McCann (2020) | Observational without control | England | Adopted children | approx 5-8 years | 51 couples | Time-limited psychodynamic therapy for couples | none | The CORE-OM; the Quality of Marriage Index; the Strengths and Difficulties Questionnaire (SDQ); the Experience of Service Questionnaire (ESQ) |
| Prout et al. (2019) | Observational without control | USA | ODD | 5-9 | 3 | Regulation-focused psychotherapy for children (RFP-C) | none | The Kiddie-Schedule for Affective Disorders and Schizophrenia–Present/Lifetime (K-SADS-PL); Child Behavior Checklist (CBCL); the Wechsler Abbreviated Scale of Intelligence (WAIS); the Oppositional Defiant Disorder Rating Scale (ODD-RS); the Emotion Regulation Checklist (ERC) |
| Ryan & Jenkins (2020) | Observational without control | UK | Mixed Diagnoses | Not Specified | -- | The Bridge in Schools program | none | Strengths and Difficulties Questionnaire (SDQ); Child Global Assessment Scale (CGAS); Child Outcome rating Scale (CORS) |
| Salzer et al. (2018) | RCT | Germany | Social Anxiety Disorder | 14-20 | 107 | Psychodynamic Psychotherapy | Waiting List | The Liebowitz Social Anxiety Scale for Children and Adolescents (LSAS-CA); the Social Phobia Anxiety Inventory (SPAI) |
| Stefini et al. (2017) | RCT | Germany | Bulimia Nervosa | 14-20 | 81 | Psychodynamic Psychotherapy | CBT | Eating Disorder Examination (EDE); the SCID-I28 and SCID-II29 for the DSM-IV; The Symptom Check List (SCL-90-R) |
| Strangio et al. (2017) | Quasi-experimental | Italy | Feeding and eating disorder, and comorbid Addictive and/or Impulse Control Disorders. | 13–18 | 26 | Psychodynamic Psychotherapy | none | Eating Disorder Inventory-3 (EDI); the Symptom Checklist-90 (SC-90); the Barratt Impulsiveness Scale-11; the Dissociative Experiences Scale (DES); the Global Assessment of Functioning (CGAS); the Semi-structured Interview for DSM-IV Axis II (SCID-II); the Childhood Trauma Questionnaire (CTS) - short form |
| Weitkamp et al. (2017) | Quasi-experimental | Germany | Externalising Disorders | 4-21 | 93 | Psychoanalytic Psychotherapy | Waiting List with supportive bridging sessions | Child Behaviour Checklist/Youth Self Report (CBCL/YSR); the Schedule for Affective Disorders and Schizophrenia for School-Age Children (K-SADS) |
| Weitkamp et al. (2018) | Quasi-experimental | Germany | Anxiety | 4-21 | 88 | Psychoanalytic Psychotherapy | Waiting List with supportive bridging sessions | The Screen for Child Anxiety Related Emotional Disorders (SCARED); K-SADS-P interview; Treatment fidelity checklist. |

**Table 3. All studies, organised by presenting problem***

*Some papers appear twice in this table, where secondary analysis has focused on a subset of the original sample, and this subset falls into a different ‘presenting problem’ category than the original sample of the overall study.

| Study Design | Definition |
| --- | --- |
| Observational | *A naturalistic study with no control group* |
| Observational with control | *The study includes a paired/matched control, such as a community sample or TAU, but with no allocation of study participants to this control group* |
| Quasi-experimental | *The study employed quasi-experimental methods, using a control group with non-randomised allocation of participants to each treatment arm* |
| RCT | *Randomised Control Trial, with random allocation of participants to each treatment arm* |

| Presenting Problem | Papers | Study Design |
| --- | --- | --- |
| Mixed Emotional Disorders | Target et al. 2002; Haslam 2008. | Observational |
|  | Muratori et al. 2002; Muratori et al. 2003; Muratori et al. 2005. | Quasi-experimental |
|  | Smyrnios & Kirby, 1993. | RCT |
|  | Sinha & Kapur, 1999. | RCT |
|  | Target & Fonagy, 1994a. | Observational (secondary analysis) |
|  | Salzer et al. 2014; Cropp et al. 2019 | RCT |
| Depression | Goodyer et al., 2017; Goodyer, et al. 2016; Aitken et al. 2020; Davies et al. 2020; O'Keefe et al. 2019; O'Keefe et al. 2020; Reynolds et al. 2020. | RCT |
|  | Trowell et al. 2007, 2003, 2009, 2010; Kolaitis et al. 2014; Garoff et al. 2011. | RCT |
|  | Weitkamp et al. 2014. | quasi-experimental |
|  | Lindqvist et al. 2020. | RCT |
|  | Horn et al. 2005 | Observational (secondary analysis) |
| Anxiety | Salzer et al. 2018 | RCT |
|  | Weitkamp et al. 2018 | quasi-experimental |
|  | Göttken et al. 2014 | quasi-experimental |
|  | Milrod et al. 2013 | Observational |
|  | Kronmuller et al. 2005. | Observational (secondary analysis) |
|  | Muratori et al. 2005. | Quasi-experimental (secondary analysis) |
| *OCD* | Apter et al. 1984. | Controlled observation study |
| *Self Harm* | Griffiths et al. 2019. | RCT |
|  | Rossouw & Fonagy, 2012. | RCT |
| Eating Disorder | Chirico et al. 2019. | Observational |
|  | Strangio et al. 2017. | Observational with control |
|  | Stefini et al. 2017 | RCT |
|  | Lock et al. 2010. | RCT |
|  | Robin et al. 1995; Robin et al. 1999. | RCT |
|  | Vilvisk & Vaglum, 1990. | Observational |
| Externalising Disorders | Edginton et al. 2018. | RCT |
|  | Prout et al. 2019. | Observational |
|  | Weitkamp et al. 2017. | quasi-experimental |
|  | Eresund, 2007. | Observational |
|  | Fonagy & Target, 1994 | Observational (secondary analysis) |
|  | Laezer, 2015. | Observational with control |
|  | Jordy & Gorodscy, 1996. | Observational with control |
|  | Winkelmann et al. 2005. | Observational (secondary analysis) |
| Mixed Diagnostic Groups | Gatta et al., 2019. | Observational |
|  | Halfon & Bulut, 2017; Halfon, Cavdar, & Yilmaz, 2019; Halfon, Ozsoy, & Cavdar, 2019. | Observational |
|  | Krischer, 2020. | Observational with control |
|  | Ryan & Jenkins, 2020. | Observational |
|  | Bury et al. 2007 | Observational |
|  | Barbre, 2005 | Observational |
|  | Deakin & Nunns, 2009. | Observational with control |
|  | Odhammar, 2011; Carlberg et al. 2009. | Observational |
|  | Schachter & Target; 2009; Midgley & Target, 2005; Midgley et al. 2006. | Observational with control |
|  | Tishby et al. 2007. | Observational with control |
|  | Tonge et al. 2009. | Observational with control |
|  | Urwin, 2007. | Observational |
|  | Edlund et al. 2014. | Observational |
|  | Edlund & Carlberg, 2016. | Observational |
|  | Emanuel et al. 2014. | Observational |
|  | Gatta et al. 2016. | Observational |
|  | Krischer et al. 2013. | Observational |
|  | Ryynänen et al. 2015. | Observational with control |
|  | Seiffge-Krenke & Nitzko, 2011. | Observational |
|  | Atzil-Slonim et al. 2011; Atzil-Slonim et al. 2013 | Observational with control |
|  | Stefini et al. 2013. | Observational |
|  | Sugar & Berkovitz, 2011a. | Observational |
|  | Petri & Thieme, 1978. | Observational |
|  | Szapocznik et al. 1989. | RCT |
|  | Fahrig et al.1996. | Observational |
|  | Baruch, 1995; Baruch et al., 1998, Baruch & Fearon, 2002; Baruch & Vrouva, 2010. | Observational |
|  | The Heidelberg Study: Kronmuller et al., 2002, 2005, 2010; Horn et al., 2005; Windaus, 2005, Stefini et al. 2009. | quasi-experimental |
|  | Anna Freud Retrospective Study: Fonagy & Target, 1994; Target & Fonagy, 1994a, 1994b; Fonagy & Target, 1996 | Observational |
|  | Levy, 2018. | Observational |
| Developmental Disorders | Heinicke, 1965; Heinicke & Ramsay-Klee, 1986. | controlled observation study |
|  | Zelmann et al. 1985. | Observational |
| *ASD* | Enav et al. 2019. | quasi-experimental |
| Emerging Personality Disorders | Hauber et al. 2017. | Observational |
|  | Schenk et al. 2019. | Observational |
|  | Beck et al., 2020; Jørgensen et al. 2020. | RCT |
|  | Salzer et al. 2014. | Observational |
|  | Sugar, & Berkovitz, 2011b. | Observational |
|  | Bo et al. 2017. | Observational |
|  | Chanen et al. 2008. | RCT |
|  | Bo et al., 2019. | Observational |
| Adoption/Foster Care | Midgley et al. 2018. | Observational |
|  | Midgley et al. 2019. | RCT |
|  | Polek et al., 2020. | Observational |
|  | Clausen et al., 2012. | Observational |
|  | Lush et al. 1991; Boston & Lush, 1994; Lush et al. 1998; Boston et al. 2009. | controlled observation study |
| Trauma/Abuse | Heede et al. 2009. | Observational |
|  | Gilboa-Schechtman et al. 2010. | RCT |
|  | Trowell et al., 2002 | RCT |
| Parental Conflict | Hertzman et al. 2017. | RCT (feasibility) |
| Domestic Violence | Bernstein et al. 2019. | RCT |
|  | Pernebo et al. 2018. | quasi-experimental |
| Physical Illness | Balottin et al. 2014. | RCT |
|  | Moran & Fonagy, 1987; Fonagy & Moran 1990; Moran et al. 1991. | quasi-experimental |

**References** – All papers included in the review

Aitken, M., Haltigan, J. D., Szatmari, P., Dubicka, B., Fonagy, P., Kelvin, R., ... & Goodyer, I. M. (2020). Toward precision therapeutics: general and specific factors differentiate symptom change in depressed adolescents. *Journal of Child Psychology and Psychiatry*. *61*(9), 998-1008

Apter, A., Bernhout, E. and Tyano, S. (1984). Severe obsessive compulsive disorder in adolescence: a report of cases. *Journal of Adolescence*, *7*(4), 349–58.

Atzil-Slonim, D., Shefler, G., Dvir Gvirsman, S., & Tishby, O. (2011). Changes in rigidity and symptoms among adolescents in psychodynamic psychotherapy. *Psychotherapy Research, 21*(6), 685-697.

Atzil-Slonim, D. A., Shefler, G., Slonim, N., & Tishby, O. (2013). Adolescents in psychodynamic psychotherapy: Changes in internal representations of relationships with parents. *Psychotherapy Research, 23*(2), 201-217.

Balottin, U., Ferri, M., Racca, M., Rossi, M., Rossi, G., Beghi, E., Chiappedi, M. & Termine, C. (2014) Psychotherapy versus usual care in pediatric migraine and tension-type headache: a single-blind controlled pilot study. *Italian Journal of Pediatrics, 40*(6): 1–7.

Barbre, J. (2005). The Harlem Family Institute: evaluation and discussion of HFI’s school- based psychotherapeutic treatment with children and families. Unpublished manuscript.

Baruch, G. (1995). Evaluating the outcome of a community-based psychoanalytic psychotherapy service for young people between 12–25 years old: work in progress. *Psychoanalytic Psychotherapy, 9*(3): 243–67.

Baruch, G. & Fearon, P. (2002). The evaluation of mental health outcome at a community-based psychodynamic psychotherapy service for young people: a 12-month follow-up based on self-report data. *Psychology and Psychotherapy*, 75: 261–78.

Baruch, G. & Vrouva, I. (2010). Collecting routine outcome data in a psychotherapy clinic for young people: findings from an ongoing study. *Child and Adolescent Mental Health, 15*(1): 30–6.

Baruch, G., Fearon, P., & Gerber, A. (1998). ‘Evaluating the outcome of a community–based psychoanalytic psychotherapy service for young people: one year repeated follow up.’ In: *Rethinking Clinical Audit*, Davenhill, R. & Patrick, M., Eds. London: Routledge, pp 157-182

Beck, E., Bo, S., Jørgensen, M. S., Gondan, M., Poulsen, S., Storebø, O. J., ... & Simonsen, E. (2020). Mentalization‐based treatment in groups for adolescents with borderline personality disorder: a randomized controlled trial. *Journal of Child Psychology and Psychiatry, 61*(5), 594-604.

Bernstein, R. E., Timmons, A. C., & Lieberman, A. F. (2019). Interpersonal violence, maternal perception of infant emotion, and child-parent psychotherapy. *Journal of family violence, 34*(4), 309-320.

Bo, S., Bateman, A., & Kongerslev, M. T. (2019). Mentalization-based group therapy for adolescents with avoidant personality disorder: Adaptations and findings from a practice-based pilot evaluation*. Journal of Infant, Child, and Adolescent Psychotherapy, 18*(3), 249-262.

Bo, S., Sharp, C., Beck, E., Pedersen, J., Gondan, M., & Simonsen, E. (2017). First empirical evaluation of outcomes for mentalization-based group therapy for adolescents with BPD. *Personality Disorders: Theory, Research, and Treatment, 8*(4), 396.

Boston, M. & Lush, D. (1994). ‘Further considerations of methodology for evaluating psychoanalytic psychotherapy with children: reflections in light of research experience’*. Journal of Child Psychotherapy,* *20*(5): 205–9.

Boston, M., Lush, D. & Grainger, E. (2009). ‘Evaluation of psychoanalytic psychotherapy with fostered, adopted and ‘in care’ children’. In Midgley, N., Anderson, J., Grainger, E., Nesic-Vuckovic, T. & Urwin, C. (eds) *Child Psychotherapy and Research New Approaches, Emerging Findings.* London: Routledge.

Bury, C., Raval, H. & Lyon, L. (2007). ‘Young people’s experiences of individual psychoanalytic psychotherapy’. *Psychology and Psychotherapy: Theory, Research and Practice*, 80: 79–96.

Carlberg, G., Thoren, A., Billstrom, S. & Odhammar, F. (2009). Children’s expectations and experiences of psychodynamic child psychotherapy. *Journal of Child Psychotherapy, 35*(2), 175–93.

Chanen, A.M., Jackson, H.J., Mccutcheon, L.K., Joyey, M., Dudgeon, P., Yuen, H.P., Germano, D., Nistico, H., Mcdougall, E., Weinstein, C., Clarkson, V., & Mcgorry, P.D. (2008). Early intervention for adolescents with borderline personality disorder using cognitive analytic therapy: randomised controlled trial. *British Journal of Psychiatry*, *193*(6), 477–84

Chirico, I., Andrei, F., Salvatori, P., Malaguti, I., & Trombini, E. (2019). The Focal Play Therapy: A Clinical Approach to Promote Child Health and Family Well-being. *Frontiers in public health*, *7*, 77.

Clausen, J. M., Ruff, S. C., Wiederhold, W. V. & Heineman, T. V. (2012). ‘For as long as it takes: relationship-based play therapy for children in foster care’. *Psychoanalytic Social Work*, *19*(1–2), 43–53.

Cropp, C., Taubner, S., Salzer, S., & Streeck-Fischer, A. (2019). Psychodynamic Psychotherapy with Severely Disturbed Adolescents: Changes in Reflective Functioning. *Journal of Infant, Child, and Adolescent Psychotherapy, 18*(3), 263-273.

Davies, S.E., Neufeld, S.A., van Sprang, E., Schweren, L., Keivit, R., Fonagy, P., Dubicka, B., Kelvin, R., Midgley, N., Reynolds, S., Target, M., Wilkinson, P., van Harmelen, A.L. & Goodyer, I.M. (2020), Trajectories of depression symptom change during and following treatment in adolescents with unipolar major depression. *Journal of Child Psychology and Psychiatry,* *61*: 565-574

Deakin, E. K. & Nunes, M. L .T. (2009). Effectiveness of child psychoanalytic psychotherapy in a clinical outpatient setting. *Journal of Child Psychotherapy, 35*(3), 290–301.

Edginton, E., Walwyn, R., Twiddy, M., Wright-Hughes, A., Tubeuf, S., Reed, S., ... & Ellis, L. (2018). TIGA-CUB-manualised psychoanalytic child psychotherapy versus treatment as usual for children aged 5–11 with treatment-resistant conduct disorders and their primary carers: results from a randomised controlled feasibility trial. *Journal of Child & Adolescent Mental Health, 30*(3), 167-182.

Edlund, J. N. & Carlberg, G. (2016). Psychodynamic psychotherapy with adolescents and young adults: Outcome in routine practice. *Clinical Child Psychology and Psychiatry*, *21*(1): 66–80.

Edlund, J. N., Thorén, A. & Carlberg, G. (2014). Outcome of psychodynamic child psychotherapy in routine practice. *European Journal of Psychotherapy and Counselling, 16*(3), 228–44.

Emanuel, R., Catty, J., Anscombe, E., Cantle, A. & Muller, H. (2014). Implementing an aim- based outcome measure in a psychoanalytic child psychotherapy service: Insights, experiences and evidence. *Clinical Child Psychology and Psychiatry, 19*:169–83.

Enav, Y., Erhard‐Weiss, D., Kopelman, M., Samson, A. C., Mehta, S., Gross, J. J., & Hardan, A. Y. (2019). A non randomized mentalization intervention for parents of children with autism*. Autism Research, 12*(7), 1077-1086.

Eresund, P. (2007). Psychodynamic psychotherapy for children with disruptive disorders. *Journal of Child Psychotherapy, 33*(2): 161–80.

Fahrig, H., Kronmuller, K. T., Hartmann, M. & Rudolf, G. (1996). Therapieerfolg analytischer psychotherapie bei kindern und jugendlichen. *Zeitschrift fur Psychosoma- tische Medizin und Psychoanalyse,* 42, 375–95.

Fitzpatrick, K.K., Moye, A., Hoste, R., Lock, J. & Le Grange, D. (2010). Adolescent focused psychotherapy for adolescents with anorexia nervosa. *Journal of Contemporary Psychotherapy, 40*(1), 31–9.

Fonagy, P. & Moran, G. S. (1990). Studies of the efficacy of child psychoanalysis. *Journal of Consulting and Clinical Psychology, 58*(6), 684–95.

Fonagy, P. & Target, M. (1994) The efficacy of psychoanalysis for children with disruptive disorders. *Journal of the American Academy of Child and Adolescent Psychiatry, 33*(1), 45–55.

Fonagy, P. & Target, M. (1996). Predictors of outcome in child psychoanalysis: a retrospective study of 793 cases at the Anna Freud Centre. *Journal of the American Psychoanalytic Association, 44*(1), 27–77.

Garoff, F. F., Heinonen, K., Pesonen, A.‐K. and Almqvist, F. (2012), Depressed youth: treatment outcome and changes in family functioning in individual and family therapy*. Journal of Family Therapy, 34*, 4-23.

Gatta, M., Miscioscia, M., Svanellini, L., Spoto, A., Difronzo, M., De Sauma, M., & Ferruzza, E. (2019). Effectiveness of brief psychodynamic therapy with children and adolescents: an outcome study. *Frontiers in Pediatrics, 7*, 501.

Gatta, M., Sisti, M., Sudati, L., Miscioscia, M. & Simonelli, A. (2016). The Lausanne Trilogue Play within the outcome evaluation in infant mental health: a preliminary report. *Research in Psychotherapy: Psychopathology, Process and Outcome*, *19*(1), 19–30.

Gilboa-Schechtman, E., Foa, E.B., Shafran, N., Aderka, I.M., Powers, M.B., Rachamin, L., Rosenbach, L., Yadin, E. & Apter, A. (2010). Prolonged exposure versus dynamic therapy for adolescent PTSD: a pilot randomized controlled trial. *Journal of the American Academy of Child and Adolescent Psychiatry, 49*(10), 980–9.

Goodyer, I. M., Reynolds, S., Barrett, B., Byford, S., Dubicka, B., Hill, J., ... & Senior, R. (2016). Effectiveness And Cost-Effectiveness Of Cognitive Behaviour Therapy And Short-Term Psychoanalytic Psychotherapy Compared With Brief Psychosocial Intervention In Maintaining Reduced Depressive Symptoms 12 months after end of treatment in Adolescents with Unipolar Major Depression (IMPACT): A Pragmatic Superiority Randomised Controlled Trial. The Lancet Psychiatry.

Goodyer, I. M., Reynolds, S., Barrett, B., Byford, S., Dubicka, B., Hill, J., ... & Senior, R. (2017). Cognitive-behavioural therapy and short-term psychoanalytic psychotherapy versus brief psychosocial intervention in adolescents with unipolar major depression (IMPACT): a multicentre, pragmatic, observer-blind, randomised controlled trial. *Health Technology Assessment (Winchester, England*), *21*(12), 1.

Göttken, T., White, L.O., Klein, A. M. & Von Klitzing, K. (2014). Short-term psychoanalytic child therapy for anxious children: a pilot study. *Psychotherapy, 51*(1), 148–58.

Griffiths, H., Duffy, F., Duffy, L., Brown, S., Hockaday, H., Eliasson, E., ... & Schwannauer, M. (2019). Efficacy of Mentalization-based group therapy for adolescents: the results of a pilot randomised controlled trial. *BMC psychiatry, 19*(1), 167.

Halfon, S., & Bulut, P. (2019). Mentalization and the growth of symbolic play and affect regulation in psychodynamic therapy for children with behavioral problems. *Psychotherapy Research, 29*(5), 666-678.

Halfon, S., Özsoy, D., & Çavdar, A. (2019). Therapeutic alliance trajectories and associations with outcome in psychodynamic child psychotherapy. *Journal of consulting and clinical psychology, 87*(7), 603.

Halfon, S., Yılmaz, M., & Çavdar, A. (2019). Mentalization, session-to-session negative emotion expression, symbolic play, and affect regulation in psychodynamic child psychotherapy. *Psychotherapy, 56*(4), 555–567.

Haslam, F. (2008). *A quantitative analysis of psychotherapy outcome in four cases of child psychotherapy*. Unpublished MSc dissertation, Anna Freud Centre/ University College London.

Hauber, K., Boon, A. E., & Vermeiren, R. (2017). Examining changes in personality disorder and symptomology in an adolescent sample receiving intensive mentalization based treatment: a pilot study. *Child and adolescent psychiatry and mental health, 11*(1), 1-7.

Heede, T., Runge, H., Storebø, O. J., Rowley, E. & Hansen, K. G. (2009). Psychodynamic milieu-therapy and changes in personality - what is the connection? *Journal of Child Psychotherapy, 35*(3): 276–89.

Heinicke, C. M. (1965). Frequency of psychotherapeutic session as a factor affecting the child’s developmental status. *Psychoanalytic Study of the Child, 20*, 42–98.

Heinicke, C. M. & Ramsay-Klee, D. M. (1986). Outcome of child psychotherapy as a function of frequency of session. *Journal of the American Academy of Child and Adolescent Psychiatry, 25*(2), 247–53.

Hertzmann, L., Abse, S., Target, M., Glausius, K., Nyberg, V., & Lassri, D. (2017). Mentalisation-based therapy for parental conflict–parenting together; an intervention for parents in entrenched post-separation disputes. *Psychoanalytic Psychotherapy, 31*(2), 195-217.

Horn, H., Geiser-Elze, A., Reck, C., Hartmann, M., Stefini, A., Victor, D., ̈Winkelmann, K. & Kronmuller, K.T. (2005). Efficacy of short term psychotherapy children and adolescents with depression. *Praxis Kinderpsychol Kinderpsychiatr, 54*(7), 578–97.

Jordy, C. F. & Gorodscy, R.C. (1996). The hyperactive child and the body: a clinical study on the origin of hyperactivity in children. *Arquivos de Neuro-Psiquiatria, 54*(4), 628–36.

Jørgensen, M. S., Storebø, O. J., Bo, S., Poulsen, S., Gondan, M., Beck, E., ... & Simonsen, E. (2020). Mentalization-based treatment in groups for adolescents with borderline personality disorder: 3-and 12-month follow-up of a randomized controlled trial. European child and adolescent psychiatry.

Kolaitis, G., Giannakopoulos, G., Tomaras, V., Christogiorgos, S., Pomini, V., Layiou-Lignos, E., Tzavara, C., Rhode, M., Miles, G., Joffe, I., Trowell, J. & Tsiantis, J. (2014). Self- esteem and social adjustment in depressed youths: a randomized trial comparing psychodynamic psychotherapy and family therapy. *Psychotherapy and Psychosomatics, 83*(4), 249–51.

Krischer, M. K., Trautmann-Voigt, S., Kaspers, S., Voigt, B., Flechtner, H. H. & Lehmkuhl, G. (2013). Effectiveness of psychodynamic psychotherapy in children and juveniles – results of a pilot study. *Zeitschrift für Kinder- und Jugendpsychiatrie und Psychotherapie, 41*(2), 87–97.

Krischer, M., Smolka, B., Voigt, B., Lehmkuhl, G., Flechtner, H. H., Franke, S., ... & Trautmann-Voigt, S. (2020). Effects of long-term psychodynamic psychotherapy on life quality in mentally disturbed children. *Psychotherapy Research, 30*(8), 1039-1047.

Kronmuller, K., Postelnicu, I., Hartmann, M., Stefini, A., Geiser-Elze, A.,Gerhold, M., Hildegard, H. & Winkelmann, K. (2005). Efficacy of psychody- namic short term psychotherapy for children and adolescents with anxiety disorders. *Praxis Kinderpsychol Kinderpsychiatr*, 54(7), 559–77.

Kronmuller, K., Stefini, A., Geiser-Elze, A., Horn, H., Hartmann, M. & Winkelmann, K. (2010). ‘The Heidelberg study of psychodynamic psychotherapy for children & adolescents’. In Tsiantis, J. And Trowell, J. (eds) *Assessing Change in Psychoanalytic Psychotherapy of Children and Adolescents.* London: Karnac.

Kronmuller, K., Victor, D., Horn, H., Winkelmann, K., Reck, C., Geiser-Elze, A. & Hartmann, M. (2002). Therapeutic relationship patterns in child and adolescent psychotherapy/Muster der therapeutischen Beziehung in der Kinder- und. Jugendlichen- Psychotherapie. *Zeitschrift fur Klinische Psychologie, Psychiatrieund Psychotherapie*, *50*(3), 267–80.

Laezer, K. L. (2015). Effectiveness of psychoanalytic psychotherapy and behavioral therapy treatment in children with attention deficit hyperactivity disorder and oppositional defiant disorder. *Journal of Infant, Child, and Adolescent Psychotherapy*, *14*(2): 111–28.

Levy, J. (2017). Relationships for Growth and Learning: Zooming in Unpacking Therapeutic Change in Group Treatment for At-Risk Preschoolers Through an Exploratory, Idiographic, and Mixed-Methods Approach. (Doctoral Thesis). Available from ProQuest Dissertations Publishing, number: 10272117.

Lindqvist, K., Mechler, J., Carlbring, P., Lilliengren, P., Falkenström, F., Andersson, G., ... & Midgley, N. (2020). Affect-Focused Psychodynamic Internet-Based Therapy for Adolescent Depression: Randomized Controlled Trial. *Journal of Medical Internet Research, 22*(3), e18047.

Lock, J., Le Grange, D., Agras, W. S., Moye, A., Bryson, S. W. & Jo, B. (2010). Randomized clinical trial comparing family-based treatment with adolescent-focused individual therapy for adolescents with anorexia nervosa. *Archives of General Psychiatry, 67*(10): 1025–32.

Lush, D., Boston, M. & Grainger, E. (1991). Evaluation of psychoanalytic psychotherapy with children: therapists’ assessments and predictions. *Psychoanalytic Psychotherapy, 5*(3), 191–234.

Lush, D., Boston, M., Morgan, J. & Kolvin, I. (1998) Psychoanalytic psychotherapy with disturbed adopted and foster children: a single case follow-up study. *Clinical Child Psychology and Psychiatry, 3*(1), 51–69.

Midgley, N. & Target, M. (2005). Recollections of being in child psychoanalysis: a qualitative study of a long-term follow-up study. *Psychoanalytic Study of the Child, 60*, 157–77

Midgley, N., Alayza, A., Lawrence, H., & Bellew, R. (2018). Adopting Minds—A mentalization-based therapy for families in a post-adoption support service: Preliminary evaluation and service user experience. *Adoption & Fostering, 42*(1), 22-37.

Midgley, N., Besser, S. J., Fearon, P., Wyatt, S., Byford, S., & Wellsted, D. (2019). The Herts and Minds study: feasibility of a randomised controlled trial of Mentalization-Based Treatment versus usual care to support the wellbeing of children in foster care. *BMC psychiatry, 19*(1), 215.

Midgley, N., Target, M. And Smith, J. A. (2006). The outcome of child psychoanalysis from the patient’s point of view: a qualitative analysis of a long-term follow-up study. *Psychology and Psychotherapy: Theory, Practice, Research, 79*, 257–69.

Milrod, B., Shapiro, T., Gross, C., Silver, G., Preter, S., Libow, A. & Leon, A.C. (2013). Does manualized psychodynamic psychotherapy have an impact on youth anxiety disorders?. *American Journal of Psychotherapy, 67*(4), 359–66.

Moran, G. & Fonagy, P. (1987). Psychoanalysis and diabetic control: a single-case study. *British Journal of Medical Psychology, 60*, 357–72.

Moran, G., Fonagy, P., Kurtz, A., Bolton, A. M. & Brook, C. (1991). A controlled study of the psychoanalytic treatment of brittle diabetes. *Journal of the American Academy of Child and Adolescent Psychiatry, 30*(6), 926–35.

Muratori, F., Picchi, L., Apicella, F., Salvadori, F., Espasa, F.P., Ferretti, D. & Bruni, G. (2005). Psychodynamic psychotherapy for separation anxiety disorders in children. *Depression and Anxiety, 21*(1), 45–6.

Muratori, F., Picchi, L., Bruni, G., Patarnello, M. & Romagnoli, G. (2003). A two-year follow up of psychodynamic psychotherapy for internalizing disorders in children. *Journal of the American Academy of Child and Adolescent Psychiatry, 42*(3): 331–9.

Muratori, F., Picchi, L., Casella, C., Tancredi, R., Milone, A. & Patarnello, M.G. (2002). Efficacy of brief dynamic psychotherapy for children with emotional disorders. *Psychotherapy and Psychosomatics, 71*(1), 28–38.

O'Keeffe, S., Martin, P., & Midgley, N. (2020). When adolescents stop psychological therapy: Rupture–repair in the therapeutic alliance and association with therapy ending. *Psychotherapy.*

O’Keeffe, S., Martin, P., Goodyer, I. M., Kelvin, R., Dubicka, B., Reynolds, S., ... & Midgley, N. (2019). Prognostic implications for adolescents with depression who drop out of psychological treatment during a randomized controlled trial. *Journal of the American Academy of Child & Adolescent Psychiatry, 58*(10), 983-992.

Odhammar, F., Sundin, E.C., Jonson, M. & Carlberg, G. (2011). Children in psychodynamic psychotherapy: changes in global functioning. *Journal of Child Psychotherapy, 37*(3), 261–279.

Pernebo, K., Fridell, M., & Almqvist, K. (2018). Outcomes of psychotherapeutic and psychoeducative group interventions for children exposed to intimate partner violence. *Child Abuse & Neglect, 79,* 213-223.

Petri, H. & Thieme, E. (1978). ‘Katamnese zur analytischen psychotherapie im kindes und Jugendalter’. *Psyche, 1*, 21–54.

Polek, E., & McCann, D. (2020). The feasibility and effectiveness of a time-limited psychodynamic couple-focused therapy for adoptive parents: preliminary evidence from the Adopting Together project. *Adoption & Fostering, 44*(1), 75-91.

Prout, T. A., Rice, T., Murphy, S., Gaines, E., Aizin, S., Sessler, D., ... & Hoffman, L. (2019). Why is it easier to get mad than it is to feel sad? Pilot study of regulation-focused psychotherapy for children. *American journal of psychotherapy, 72*(1), 2-8.

Reynolds, S., Orchard, F., Midgley, N., Kelvin, R., & Goodyer, I. (2020). Do sleep disturbances in depressed adolescents improve following psychological treatment for depression?. *Journal of Affective Disorders, 262*, 205-210.

Robin, A., Siegel, P., Moye, A., Gilroy, M., Dennis, A.B. & Sikand, A. (1999). A controlled comparison of family versus individual psychotherapy for adolescents with anorexia nervosa. *Journal of the American Academy of Child and Adolescent Psychiatry, 38*(12), 1482–9.

Robin, A., Siegel, T. & Moye, A. (1995). Family versus individual therapy for anorexia: impact on family conflict. *International Journal of Eating Disorders, 17*(4), 313–22.

Rossouw, T. I. & Fonagy, P. (2012). Mentalization-based treatment for self-harm in adolescents: a randomized controlled trial. *Journal of the American Academy of Child and Adolescent Psychiatry, 51*(12), 1304–13.

Ryan, E., & Jenkins, M. (2020). The Bridge in Schools: a psychodynamic impact model for child Mental Health In Disadvantaged Areas. *Journal Of Child Psychotherapy,* 1-21.

Ryynänen, T., Alen, M., Koivumaa-Honkanen, H., Joskitt, L. & Ebeling, H. (2015). Implementation and outcome of child psychotherapy compared with other psychiatric

Salzer, S., Cropp, C. & Streeck-Fischer, A. (2014). Early intervention for borderline personality disorder: psychodynamic therapy in adolescents. *Zeitschrift für Psychosomatische Medizin und Psychotherapie, 60*(4), 368–82.

Salzer, S., Cropp, C., Jaeger, U., Masuhr, O. & Streeck-Fischer, A. (2013). Psychodynamic therapy for adolescents suffering from co-morbid disorders of conduct and emotions in an in-patient setting: a randomized controlled trial. *Psychological Medicine, 44*, 2213–22.

Salzer, S., Stefini, A., Kronmüller, K. T., Leibing, E., Leichsenring, F., Henningsen, P., ... & Schopf, Y. (2018). Cognitive-behavioral and psychodynamic therapy in adolescents with social anxiety disorder: A multicenter randomized controlled trial. *Psychotherapy and psychosomatics, 87*(4), 223-233.

Schachter, A. & Target, M. (2009). ‘The adult outcome of child psychoanalysis: the Anna Freud Centre long-term follow-up study’. In Midgley, N., Anderson, J., Grainger, E., Nesic-Vuckovic, T., & Urwin, C. (eds) *Child Psychotherapy and Research: New Approaches, Emerging Findings*. London: Routledge.

Schenk, N., Zimmermann, R., Fürer, L., Krause, M., Weise, S., Kaess, M., ... & Schmeck, K. (2019). Trajectories of alliance ruptures in the psychotherapy of adolescents with borderline personality pathology: timing, typology and significance. *Research in Psychotherapy: Psychopathology, Process and Outcome, 22*(2).

Seiffge-Krenke, I., & Nitzko, S. (2011). Wie wirksam sind analytische und tiefenpsychologisch fundierte Langzeitpsychotherapien bei Jugendlichen? [How effective are long-term psychoanalytic treatments in adolescents? A comparison between the evaluations of the patients, their parents and their therapists]. *Zeitschrift fur Kinder- und Jugendpsychiatrie und Psychotherapie*, *39*(4), 253–264.

Sinha, U. K. & Kapur, M. (1999). Psychotherapy with emotionally disturbed adolescent boys: outcome and process study’. *National Institute of Mental Health and Neuro Sciences Journal (NIMHANS), 17*(2), 113–30.

Smyrnios, K. X. & Kirby, R. J. (1993) Long term comparison of brief versus unlimited psychodynamic treatments with children and their parents. *Journal of Consulting and Clinical Psychology, 61*(6), 1020–7.

Stefini, A., Geiser-Elze, A., Hartmann, M., Horn, H., Winkelmann, K., & Kronmüller, K. T. (2009). Bindungsstil und Therapieerfolg in der psychodynamischen Kurzzeittherapie bei Kindern und Jugendlichen [Attachment style and outcome in short-term psychodynamic psychotherapy for children and adolescents]. *Psychotherapie, Psychosomatik, medizinische Psychologie*, *59*(2), 68–74.

Stefini, A., Horn, H., Winkelmann, K., Geiser-Elze, A., Hartmann, M., & Kronmüller, K. T. (2013). Attachment styles and outcome of psychoanalytic psychotherapy for children and adolescents. *Psychopathology*, *46*(3), 192–200.

Stefini, A., Salzer, S., Reich, G., Horn, H., Winkelmann, K., Bents, H., ... & Specht, N. (2017). Cognitive-behavioral and psychodynamic therapy in female adolescents with bulimia nervosa: a randomized controlled trial. *Journal of the American Academy of Child & Adolescent* *Psychiatry, 56*(4), 329-335.

Strangio, A. M., Rinaldi, L., Monniello, G., Sisti, L. G., de Waure, C., & Janiri, L. (2017). The effect of abuse history on adolescent patients with feeding and eating disorders treated through psychodynamic therapy: Comorbidities and outcome. *Frontiers in Psychiatry, 8,* 31.

Sugar, M. & Berkovitz, I. H. (2011a) Male adolescent treatment outcome: a case series of eight men treated with psychoanalytic psychotherapy*. Adolescent Psychiatry, 1*(2), 169–78.

Sugar, M. & Berkovitz, I. H. (2011b) Treatment outcome of three female adolescents with borderline personality disorder. *Adolescent Psychiatry, 1*(1): 6–19.

Szapocznik, J., Rio, A., Murray, E., Cohen, R., Scopetta, M., Rivas-Vazquez, A., Hervis, O., Posada, V., & Kurtines, W. (1989). Structural family versus psychodynamic child therapy for problematic Hispanic boys. Journal of Consulting and Clinical Psychology, 57(5), 571–578.

Target, M. & Fonagy, P. (1994a) The efficacy of psychoanalysis for children with emotional disorders. *Journal of the American Academy of Child and Adolescent Psychiatry*, *33*: 361–71.

Target, M. & Fonagy, P. (1994b) The efficacy of psychoanalysis for children: prediction of outcome in a developmental context. *Journal of the American Academy of Child and Adolescent Psychiatry, 33*(8), 1134–44.

Target, M., March, J., Ensink, K., Fabricius, J. & Fonagy, P. (2002). ‘Prospective study of the outcome of child psychoanalysis and psychotherapy (AFC5)’. In Fonagy, P. (ed.) *An Open Door Review of Outcome Studies in Psychoanalysis (2nd edition).* London: International Psychoanalytic Association.

Tishby, O., Raitchick, I. & Shefler, G. (2007). Changes in interpersonal conflicts among adolescents during psychodynamic therapy. *Psychotherapy Research, 17*(3), 297– 304.

Tonge, B. T., Pullen, J. M., Hughes, G. C. & Beafoy, J. (2009). Effectiveness of psychoanalytic psychotherapy for adolescents with serious mental illness: 12 month naturalistic study. *Australian and New Zealand Journal of Psychiatry, 43*(5), 467–75.

Trowell, J., Joffe, I., Campbell, J., Clemente, C., Almqvist, F., Soininen, M., Koskenranta-Aalto, U., Weintraub, S., Kolaitis, G., Tomaras, V., Anastasopoulos, D., Grayson, K., Barnes, J., & Tsiantis, J. (2007). Childhood depression: a place for psychotherapy. *European Child and Adolescent Psychiatry, 16*(3), 157–67.

Trowell, J., Kolvin, I., Weeramanthri, T., Sadowski, H., Berelowitz, M., Glasser, D. & Leitch, I. (2002). Psychotherapy for sexually abused girls: psychopathological outcome findings and patterns of change. *British Journal of Psychiatry*, 180, 234–47.

Trowell, J., Rhode, M. & Hall, J. (2010) ‘What does a manual contribute to work with depressed people?’ In Tsiantis, J. & Trowell, J. (eds) *Assessing Change in Psychoanalytic Psychotherapy of Children and Adolescents.* London: Karnac.

Trowell, J., Rhode, M. And Joffe, I. (2009) ‘Children depression: an outcome research project’. In Midgley, N., Anderson, J., Grainger, E., Nesic-Vuckovic, T. & Urwin, C. (eds) *Child Psychotherapy and Research: New Approaches, Emerging Findings.* London: Routledge.

Trowell, J., Rhode, M., Miles, G. & Sherwood, I. (2003). Childhood depression: work in progress. *Journal of Child Psychotherapy, 29*(2), 147–70.

Urwin, C. (2007). ‘Revisiting ‘‘what works for whom?’’: a qualitative framework for evaluating clinical effectiveness in child psychotherapy’. *Journal of Child Psychotherapy, 33*(2), 134–60.

Vilvisk, S. O. & Vaglum, P. (1990). Teenage anorexia nervosa: a 1–9 year follow up after psychodynamic treatment. *Nordic Journal of Psychiatry, 44*(3), 249–55.

Weitkamp, K., Daniels, J. K., Baumeister‐Duru, A., Wulf, A., Romer, G., & Wiegand‐Grefe, S. (2018). Effectiveness trial of psychoanalytic psychotherapy for children and adolescents with severe anxiety symptoms in a naturalistic treatment setting. *British journal of psychotherapy, 34*(2), 300-318.

Weitkamp, K., Daniels, J. K., Romer, G., & Wiegand-Grefe, S. (2017). Psychoanalytic psychotherapy for children and adolescents with severe externalising psychopathology: An effectiveness trial. *Zeitschrift für Psychosomatische Medizin und Psychotherapie, 63*(3), 251-266.

Weitkamp, K., Daniels, J.K., Hofmann, H., Timmermann, H., Romer, G. & Wiegand-Grefe, S. (2014). Psychoanalytic psychotherapy for children and adolescents with severe depressive psychopathology: preliminary results of an effectiveness trial. *Psychotherapy, 51*(1), 138–47.

Windaus, E. (2006). The position and outcome of research in Europe in child and adolescent section. Paper presented at the European Federation of Psychoanalytic Psychotherapy Conference, Berlin.

Winkelmann, K., Hartmann, M., Neumann, K., Hennch, C., Reck, C., Victor, D.,̈horn, H., Uebel, T. & Kronmuller, K.T. (2000). Stability of therapeutic outcome after child and adolescent psychoanalytical therapy. *Praxis Kinderpsychol Kinderpsy-chiatr*, *49*: 315–28.

Zelmann, A.B., Samuels, S. & Abrams, D. (1985). I.Q. Changes in young children following intensive long-term psychotherapy. *American Journal of Psychotherapy, 39*(2), 215–7.
